# How do gender transformative interventions reduce adolescent pregnancy in low- and middle-income countries: a realist synthesis

**DOI:** 10.7189/jogh.15.04102

**Published:** 2025-04-04

**Authors:** Shruti Shukla, Aishwarya Kharade, Ines Böhret, Manzura Jumaniyazova, Sarah R Meyer, Ibukun-Oluwa Omolade Abejirinde, Yulia Shenderovich, Janina Steinert

**Affiliations:** 1 School of Social Sciences and Technology, Technical University of Munich, Munich, Germany; 2 Institute for Medical Information Processing, Biometry, and Epidemiology, Ludwig-Maximilian-Universität München, Munich, Germany; 3 Program for Appropriate Technology in Health (PATH), Delhi, India; 4 Heidelberg Institute of Global Health, Heidelberg, Germany; 5 Division of Social & Behavioral Health Sciences, University of Toronto Dalla Lana School of Public Health & Women’s College Hospital Research Institute, Toronto, Canada; 6 Institute for Better Health, Trillium Health Partners, Mississauga, Canada; 7 Wolfson Centre for Young People’s Mental Health, Cardiff University, Cardiff, UK; 8 Centre for Development, Evaluation, Complexity and Implementation in Public Health Improvement, School of Social Sciences, Cardiff University, Cardiff, UK

## Abstract

**Background:**

Adolescent pregnancy poses a significant health challenge for girls aged 15–19 in low- and middle-income countries. While gender transformative interventions (GTIs) aim to address this issue, a substantial research gap exists concerning the underlying mechanisms contributing to their success. This study employs a realist synthesis approach to systematically investigate how, why, for whom, and in what contexts GTIs effectively reduce adolescent pregnancy.

**Methods:**

A five-step realist review examined literature from four databases and five organisational repositories, including published and grey literature. The review focused on GTIs for adolescents aged 10–19 in low- or middle-income countries. Narrative synthesis and realist analysis were used to develop context–mechanism–outcome configurations.

**Results:**

The review analysed 28 documents covering 14 interventions and proposing eight programme theories across three settings. In the school, creating a supportive environment to foster positive social norms and providing a safe space was emphasised. Comprehensive sexual health education to promote critical thinking, knowledge retention, and goal setting was one of the key strategies. Empowering boys to adopt positive gender norms for behaviour change was also identified. In the health facility, providing a safe, supportive, and confidential environment for accessing services, as well as using digital health apps to empower adolescents in sexual reproductive health, were key. In the community, empowering girls through life skills and economic support and involving community members to foster stronger interpersonal bonds and a gender-positive environment were highlighted. These interventions led to increased contraceptive use, delayed marriage, and reduced adolescent pregnancy.

**Conclusion:**

This realist synthesis proposes eight nuanced programme theories of successful GTIs, providing essential insights for developing, implementing, and improving future programmes. These findings offer a foundation for effective strategies to mitigate adolescent pregnancy in diverse socio-cultural contexts.

**Registration:**

PROSPERO: CRD42023398293.

Adolescence is a crucial stage of human development characterised by significant physical, emotional, and social changes. In low-income and middle-income countries (LMICs), where approximately 21 million girls aged 15–19 years become pregnant each year, and nearly 12 million girls give birth annually, investing in the health and well-being of adolescents is imperative to ensure improved health outcomes through their life-course [1,2].

A recent study examining the prevalence of adolescent motherhood using Demographic and Health Surveys data from 74 LMICs between 1990–2018 highlights substantial regional variation in adolescent pregnancy rates [3]. Sub-Saharan Africa (SSA) shows the highest prevalence, but even within this region, rates vary significantly – from 40.4% in Niger, where early marriage is widely practised, to 7.3% in Rwanda, which has seen successful national policies focused on adolescent sexual reproductive health (SRH) and rights [3-5]. In South and Southeast Asia, as well as Latin America and the Caribbean, adolescent pregnancy rates have declined. However, progress remains slow in high-burden countries such as Bangladesh, where cultural norms around early marriage persist [6], and in parts of Latin America like Guatemala and Honduras, where socioeconomic challenges like widespread poverty, scarcity of economic opportunity, high rates of school dropout and unmet need for contraception compound the issue [3,7]. Within countries, disparities are particularly marked among the most disadvantaged groups of adolescent girls [3]. Despite these variations, adolescents remain an often-neglected group in public health policies in LMICs, where maternal and child health initiatives tend to focus broadly on women of reproductive age without targeted strategies for adolescents [8].

Adolescent pregnancy, defined as the occurrence of pregnancy in girls under the age of 20, poses multifaceted challenges, affecting the health, education, and economic prospects of young mothers and their offspring [9]. Harmful gender norms like cultural expectations around childbearing and motherhood, beliefs and expectations around men’s and women’s sexuality, stigma around premarital sex, and financial dependence on partners or guardians are some of the key drivers of adverse outcomes like adolescent pregnancy and early marriage [10-13]. These norms often intersect with factors like socioeconomic status, religion and rurality, intensifying the vulnerabilities of certain groups of adolescents. For instance, recent studies in the SSA have shown that adolescent maternity and marriage were higher in the poorest wealth quintile, among girls in rural areas and Muslim communities. This was due to the lack of access to quality education and reproductive health services and norms around dowry and religious morality [14-16]. This insight is further corroborated by a study in India that highlights cultural factors such as early arranged marriages and gender norms may underlie the pathways to adolescent motherhood [10].

Adolescent pregnancy is associated with increased risks of complications such as eclampsia and puerperal endometritis, as well as adverse outcomes for newborns, including preterm delivery and low birth weight [17,18]. Worldwide, in comparison to all women and girls, fewer adolescent girls and young women receive maternal health coverage, including antenatal care, skilled delivery or postnatal care [19]. In addition to these health implications, pregnant adolescents also face several social consequences, including intimate partner violence, early/forced marriage, and school drop-out, perpetuating a cycle of poverty and inequality that could have generational effects [10,20-23]. Furthermore, harmful gender norms also create disparities in accessing health services and information, limit control over family planning decisions, and perpetuate the lower social status of women and girls in society [24].

Addressing harmful gender norms has emerged as one of the key approaches in programmes aimed at reducing gender inequality and improving adolescent health. One prominent strategy is the implementation of gender transformative interventions (GTIs), which seek to challenge and transform harmful gender norms by addressing bias within individuals, families, communities, and institutions while fostering positive interactions between men and women to promote health for all [25]. Recent systematic reviews on GTIs have underscored their potential to enhance knowledge, attitudes, and behaviours related to health among adolescents [26,27]. Specifically concerning adolescent pregnancy, several systematic reviews suggest that interventions focusing on knowledge and skills development, contraceptive promotion, conditional cash transfers, and efforts to lower educational barriers could play a role in reducing adolescent pregnancies [28-30]. However, evidence supporting these programmes is often outdated, particularly in light of rising adolescent pregnancy rates during the COVID-19 pandemic. Despite these promising findings, five critical gaps persist in the application of GTIs to address adolescent pregnancy and its underlying factors. First, research by Levy et al. [26] found that only five out of 61 evaluation studies measured changes in the incidence of unwanted or unintended pregnancies, which highlights the dearth of information on this topic. Second, McAteer et al. [27] highlighted that only 8% of the GTIs on SRH engaged men/boys. This raise concerns that without involving men and boys, these interventions are unlikely to effectively address structural issues and challenge the patriarchal norms that perpetuate gender inequalities. Third, the predominance of quantitative studies which focus on girls and a lack of discussions on potential differential mechanisms of change limit our understanding of how GTIs may differ in their impacts on girls and boys [26,27]. Fourth, most studies of GTIs lack measures on potential changes in gender norms, thereby hindering the ability to discern how these changes may contribute to positive health outcomes [31,32]. Lastly, most GTIs do not report conducting needs assessments or formative research, thereby limiting insights into the sociocultural context necessary for fostering positive shifts in harmful gender norms [33].

In this realist review, we synthesise evidence from a broad range of data sources to identify how intervention and implementation characteristics such as location, intervention provider, and resources impact the outcomes. Based on the findings, we highlight potential programme theories that can enable an understanding of the mechanisms underlying intervention success or failure and effectiveness for different participant groups. The protocol for this review was prospectively registered on PROSPERO (Ref: CRD42023398293) and has been published [32].

## METHODS

Realist synthesis is a theory-driven and qualitative evidence-synthesis methodology that is different from meta-analysis or other statistical techniques conventionally used to synthesise quantitative data [34]. Unlike traditional reviews that usually focus on whether an intervention works, a realist review is focused on uncovering how an intervention works, whom it works for and in what circumstances it works [35]. It does so by targeting the analysis process on context-mechanism-outcome configurations (CMOCs) [36]. Definitions of these concepts are presented in **Table 1**. We present the CMOCs as *if…then…because* statements aiding intuitive understanding [39].

**Table 1:** Definition of realist concepts

Concept	Definition/description
Gender Transformative Interventions (GTI)	Refers to gender transformative interventions that explicitly examine and address power relations associated with men and women and boys and girls in programmes and interventions [37].
Context (C)	Refers to the various pre-existing conditions in which the interventions were implemented. We examine context broadly in terms of the social and economic environment as well as in three settings: i) school, ii) health facility, and iii) community
Mechanism (Mechanism Resources (M-Res) and Mechanism Reasoning (M-Rea))	Refers to the causal forces behind the intervention, which can explain the (un)intended outcome of its use. We disaggregate mechanisms into resources (M-Res) and reasoning (M-Rea). Resources refer to strategies or components introduced by the interventions, and reasoning is the behavioural response triggered in the participants by introducing the resources in a given context [38].
Outcome (O)	Refers to the changes brought about by the intervention. For this review, the main outcome of interest is adolescent pregnancy, but we also explore more proximal outcomes like child marriage, contraceptive use, continuation of education, and risky sexual health behaviours.

**Figure 1:**
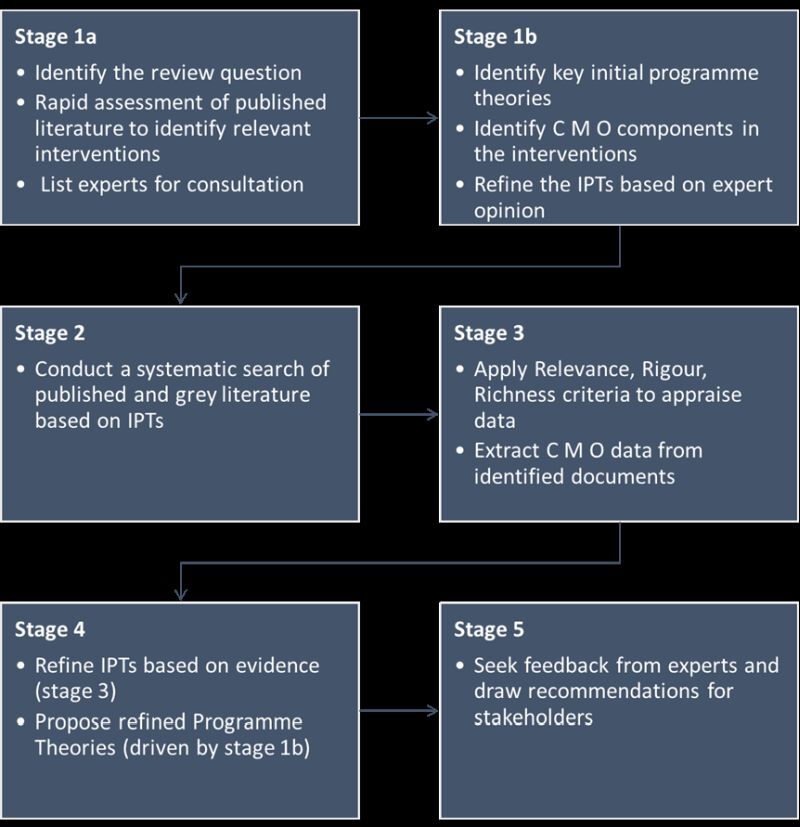
Realist review process. CMO – context-mechanism-outcome, IPTs – initial programme theories.

A realist synthesis begins with initial programme theories (IPTs), formulated by reviewing existing literature and through expert consultations. Initial programme theories are then tested iteratively against data collected and synthesised during the systematic search. Context-mechanism-outcome configurations found in the extracted data yield insights that inform further refinement of IPTs into programme theories (PTs). Programme theories explain how and under what conditions interventions are effective. To conduct this synthesis, we adapted the iterative process described by Pawson et al. (2005) [34] described in **Figure 1**.

### Stage 1a and 1b assessment of published evidence and initial programme theory development

We rapidly assessed seven systematic reviews on GTIs targeting adolescent health [26,29,40-44]. Eighteen studies included in these reviews focused on adolescent pregnancy as an outcome (Table S1 in the **Online Supplementary Document**). We developed a codebook based on CMOCs to facilitate data extraction from these studies (Table S2 in the **Online Supplementary Document**) [45,46]. Next, we developed IPTs on how GTIs work to prevent adolescent pregnancy by gathering data encompassing study context, intervention specifics, strategies, implementation methods, diverse outcomes, and plausible mechanisms from the 18 selected studies. Subsequently, we organised the extracted information into a framework incorporating CMO components (Figure S3 in the **Online Supplementary Document**). Based on the framework, eight IPTs were proposed (Table S4 in the **Online Supplementary Document**). Next, input was sought from seven experts in SRH, programme design, realist evaluation design, and adolescent health on the proposed IPTs (Table S5 in the **Online Supplementary Document**). Based on consultations, the IPTs underwent further revisions. Further details are included in the published protocol [32].

### Stage 2 systematic search

Informed by the 18 studies and the CMO framework, we applied a systematic search strategy in November and December 2023 (Table S6 in the **Online Supplementary Document**) across four databases (Pubmed, OVID, Clarivate, EBSCOhost) and five organisation repositories for grey literature (World Health Organization, United Nations Population Fund, Guttmacher, Population Council, United States Agency for International Development) to identify additional evidence to refine the IPTs. We also completed forward and backward citation tracing, contacted authors in case of missing information and repeated the search strategy in February 2024 to identify studies that may have been left out in the initial search. Further details are included in the published protocol [32] and the supplementary files.

### Stage 3 data appraisal and extraction

#### Appraisal of evidence

Four reviewers (SS, MJ, IB and AK) screened the records for relevance by title, abstract, and full text. All conflicts were resolved by a third reviewer (JS or YS). Selected full-text articles were appraised based on their richness and rigour. Richness explains how an intervention is expected to work by discussing potential theory of change, conceptual frameworks or causal pathways [47]. Based on the intervention design information, we rated the selected articles on a low to medium to high scale. Rigour measures the trustworthiness and credibility of the data source and the methods used for analysis [47]. Again, we devised a three-point scale ranging from low to high based on Joanna Briggs Institute’s (JBI) critical appraisal tools [48]. No article was excluded or included based on just one criterion but on the overall value added to the research question (Table S7 in the **Online Supplementary Document**). This relevance, richness, and rigour (RRR) assessment was performed to determine to what extent the quality and level of insights presented in the included studies could contribute to PT development [49,50]. Further details are included in the supplement files.

#### Data extraction

The data extraction form (Table S8 in the **Online Supplementary Document**) was piloted by four reviewers using two full-text records, one with a high RRR score and another with a low score. The tool was modified based on the pilot. It included information on the publication and study (publication details, geographic location, sample size, aim of the research study), intervention (name, strategy, components, location, frequency, adaptation from another intervention, implementors, quality via process evaluation), context (setting, participant characteristics, and setting, *i.e*. individual, family, community, school, or policy), mechanism (resources and reasoning), outcomes (SRH outcomes, adolescent pregnancy and main findings), measures of empowerment and gender norms, relevant IPT from stage 1, and decision-making notes. We extracted data for all included studies.

### Stage 4 development of refined programme theories

In the fourth stage, the extracted data was organised as *if…then…because* statements representing the CMOCs for each intervention. Three authors revised these statements for internal consistency. Next, the IPTs proposed in Stage 1b were mapped to the CMOCs proposed in Stage 3 to confirm, revise or refute them. The final set of confirmed CMOCs was used for further analysis. The full review team provided feedback on the proposed CMOCs in an online meeting. Similar CMOCs were combined to create PTs through an iterative process of testing them against each other to distinguish between different contexts and mechanisms. Each step of this procedure was discussed between the review team members. During this process, we referred to substantive theories from the women’s empowerment, intimate partner violence, social norms, and behavioural change literature to further refine the theories. Please refer to the supplement files for more details.

### Stage 5 expert feedback

In the last stage, the refined programme theories were presented to two expert groups comprising 30 individuals and five individuals for feedback. This included discussing a set of guiding questions on the formulation of CMOCs, relevance, theoretical underpinnings, and applicability of proposed PTs to interventions focusing on adolescent pregnancy and other SRH outcomes (Table S9 in the **Online Supplementary Document**). We report our findings using the Realist And Meta-narrative Evidence Syntheses Evolving Standards (RAMESES) (Table S10 in the **Online Supplementary Document**) [51].

## RESULTS

### Scope of the evidence

**Figure 2:**
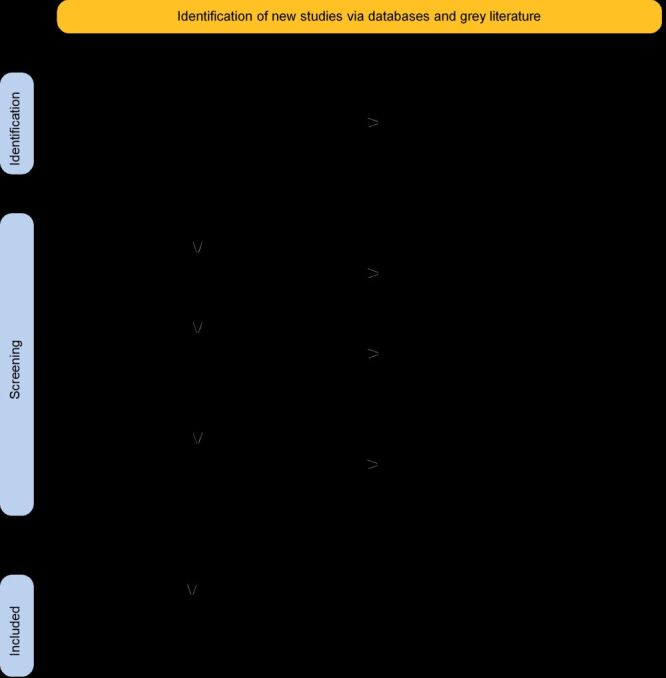
Systematic search process.

We identified 12 857 records from scientific databases and grey literature. After initial title/abstract review, 160 records were selected for full-text screening and 33 for final inclusion (**Figure 2**). Of the 33, five records had been previously included in forming IPTs and were not reanalysed [52-56]. The remaining 28 records were deemed eligible, with 19 ranking high, seven medium and two low on the RRR assessment metric [57-84]. More details on the process can be found in the previously published protocol [32].

The 28 included documents comprised 15 peer-reviewed articles [57,60-63,65,67,68,70,74,76,77,79,81,82], five baseline or endline assessment reports [72,73,75,78,83], five protocols [64,66,69,71,84], two policy briefs [58,80], and one blog post [59], describing 14 GTIs targeting adolescent pregnancy. Interventions conceptualised adolescent pregnancy similarly by either documenting self-reported pregnancies or births that had occurred before the age of 19. The majority of documents mentioned (n = 14) using experimental designs [57-59,61,64,68-72,76-78,84], followed by cross-sectional (n = 5) [66,79,81-83], longitudinal (n = 4) [67,73,74,80], mixed methods (n = 4) [62,63,65,75], and qualitative designs (n = 1) [60].

Geographically, 12 interventions were based in SSA, one in South Asia, and one in East Asia (**Table 2**). Commonly used intervention strategies included economic strengthening (*e.g.* cash transfer, material incentives), financial education (*e.g.* on saving, microcredit, business), life-skills training (*e.g.* negotiation, conflict resolution, leadership, communication), comprehensive sexual health education (CSE), health services and products provision, gender or social norm change, and school strengthening or promotion of education. Six interventions targeted only girls, seven targeted both girls and boys and one targeted only boys (**Table 2**).

**Table 2:** Details of gender transformative interventions

Intervention name	Region	Country	Target group	Age group (years)	Gender attitudes or norm change as an outcome variable	Empowerment as an outcome variable	Intervention status and impact	Records
Adolescent Girls Empowerment Programme (AGEP)	Sub-Saharan Africa	Zambia	Girls only	10–19	Included examples: girls who hold positive gender normative beliefs and acceptability of intimate partner violence	Included examples: social safety nets and self-efficacy	Completed. Overall, there is no effect on adolescent pregnancy. However, in communities with high premarital sex and for illiterate girls, there was a positive effect in delaying pregnancies by four and nine percentage points.	[57,63,71,72,77,78]
Comprehensive Sexuality Education (CSE)	East Asia	China	Girls and boys	15–24	Included examples: attitude toward premarital sex at baseline, forced partner into sex	Included example: engaged in sexual negotiation before intercourse	Completed. No effect on adolescent pregnancy.	[74]
CSE – Health Facility Linkages (CSE-HFL)	Sub-Saharan Africa	Zambia	Girls and boys	10–19	Included, but not reported	Included, but not reported	Completed. The intervention had a positive effect in reducing adolescent pregnancy. CSE-HFL showed a significant decline of in-school pregnancies. Arm 2 recorded only 0.74% pregnancies at endline (*P* < 0.001), and arm 3, recorded 1.34% pregnancies (*P* < 0.001)	[61]
CyberRwanda	Sub-Saharan Africa	Rwanda	Girls and boys	12–19	-	-	Ongoing	[65]
Determined, Resilient, Empowered, AIDS-free, Mentored and Safe (DREAMS)	Sub-Saharan Africa	South Africa	Girls only	12–19	Included but not reported example: relationship power	-	Completed. No effect on adolescent pregnancy	[66,79,81-83]
Education, Cash Transfer, and Health Risk (SIHR)	Sub-Saharan Africa	Malawi	Girls only	13–22	-	Included examples: self-esteem, social participation, preferences for child education, and aspirations	Completed. Adolescent pregnancy was reduced after two years in the conditional cash transfer (CCT) arm, but the effects disappeared after five years. Baseline dropout girls had reductions of 5.7, 8.1, and 4.0 percentage points for being ever pregnant at rounds two, three and four. Among CCT beneficiaries a reduction of more than 10% in live births was observed.	[76]
Economic Strengthening and HIV prevention (ES-HIV)	Sub-Saharan Africa	South Africa	Girls and boys	14–17	Included gender norms but not reported	Included examples: economic knowledge, self-esteem, and participation in household budgeting	Completed. Adolescent pregnancy increased over time in all groups with 2.3% of girls testing positive for pregnancy at baseline and 5.6% testing positive for pregnancy at endline.	[68]
If I were Thabo		South Africa and Lesotho	Boys targeted but girls are included	13–18	-	-	Ongoing	[59,60,84]
In their Hands (t-safe) programme (ITH)	Sub-Saharan Africa	Kenya	Girls only	15–19	-	Included example: decision-making on contraceptive use and autonomy	Completed. Reduction in adolescent pregnancy. At endline, 14.4% of all respondents reported pregnancy, compared to 31.8% at baseline.	[62,75]
Keeping Girls in School (KGIS)	South Asia	Bangladesh	Girls only	12–19	Included example: perceptions on gender rights and gender equitable norms	Included examples: mobility, social solidarity, economic aspiration, affiliation with clubs, access to technology and mass media,	Completed. Reduction in adolescent pregnancy. Ever conceived difference-in-differences (DiD) -6.5. Currently pregnant DiD 1.3	[73,80]
Research Initiative to Support the Empowerment of Girls (RISE)	Sub-Saharan Africa	Zambia	Girls and boys	13–15	Included examples: perceived community norms around early marriage, education for girls, and use of contraceptives	Included example: self-employment in girls	Ongoing	[64]
Sugar daddy	Sub-Saharan Africa	Cameroon	Girls and boys	13–14	-	-	Completed. Reduction of adolescent pregnancy in rural areas only. No effect in urban areas. In rural areas, interventions reduced the incidence of unprotected sex and hence the likelihood of having started childbearing within one year of the intervention by 2.4–4.6 percentage points, from an average of 9.5% in the comparison group (a 25–48% decrease).	[58]
Sista2Sista	Sub-Saharan Africa	Zimbabwe	Girls only	10–19	-	-	Completed. Reduction in adolescent pregnancy by 62.0% only for participants who completed all 40 Sista2Sista exercises. For others, no effect.	[67]
Yathu Yathu (‘For us, by us’)	Sub-Saharan Africa	Zambia	Girls and boys	10–19	-	-	Completed. No effect on adolescent pregnancy.	[69,70]

All interventions included adolescents with varying age ranges. Six interventions measured changes in gender attitudes or norms, while eight reported measures of female empowerment (**Table 2**). Reported outcomes included learning and school performance, child marriage, contraceptive use, and SRH outcomes, including adolescent pregnancy (our main outcome), number of partners, medical tests and counselling, and live births. Detailed information on the interventions is included in Table S11 in the **Online Supplementary Document**.

### Building programme theories for the mechanisms of change

**Figure 3:**
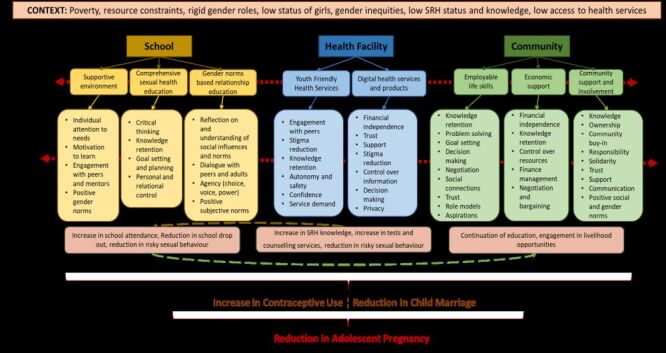
Conceptual framework of programme theories on how gender transformative interventions reduce adolescent pregnancy. SRH – sexual reproductive health.

Based on the 14 interventions, we derived 43 CMOCs and formulated eight PTs (Table S11–12 in the **Online Supplementary Document**). Three new PTs were created using CMOCs from this review, while five were refined from previously reported IPTs [32]. The programme theories and the related CMO characteristics are represented in the conceptual framework in **Figure 3**. In our review, the broad environmental context was marked by poverty, rigid gender norms, low educational and social status of girls, high gender disparities, low knowledge of SRH topics and low uptake of health services. We further organise the intervention context in three settings: school, health facility and community. Many interventions included in this review were multi-component, *i.e*. they provided inputs and were implemented in a combination of settings. Programme theories were placed in each of these settings, activating a range of mechanisms of change. These mechanisms interact with each other synergistically, as shown by the arrows, leading to the desired change of reduction in the incidence of adolescent pregnancy, our main outcome, along with other intermediate outcomes shown below. In the following section, we present evidence for the PT's success or failure under the themes of setting with details on resources, mechanisms of change and our main outcome, adolescent pregnancy.

### Programme theories for interventions delivered at the school setting targeting adolescents

#### Programme theory 1 – Supportive environment at school

If schools (Context (C) 1) are provided educational resources (*e.g*. laptops, internet, projector) for after-school sessions (Mechanism-Resources (M-res) 1), teachers receive training on interactive teaching using digital remedial curricula (M-Res2) to provide underperforming female students (C2) with grade and subject-specific tutoring tailored to address their specific needs, and local female mentors from the community are assigned to schools to discuss gender and SRH topics with female students (M-Res3).

Then, student attendance, psychosocial well-being and learning outcomes are likely to improve (Intermediate outcomes (IO)).

Because using digital educational resources and needs-specific methods can enhance the learning environment by making learning more interactive and engaging, and cater to students' needs and learning styles (Mechanism-Reasoning (M-Rea) 1). Tailored tutoring by school teachers provides girls with individualised attention and assistance which increases their confidence and motivation to learn (M-Rea2). Discussing sensitive topics with mentors will help students voice their issues and engage with each other in a supportive environment (M-Rea3). Involving local female mentors also fosters a sense of community within the school environment, providing students with additional avenues for seeking help and aiding teachers with their workload (M-Rea4). As a result, female students feel supported and motivated to learn (M-Rea5), the school observes positive social norm change (M-Rea6), and girls might continue with their education and find job opportunities, which might delay marriage and pregnancy (Outcome (O)).

The first PT is supported by three CMOCs extracted from two interventions – Keeping Girls in School (KGIS) and Determined, Resilient, Empowered, AIDS-free, Mentored and Safe (DREAMS) – spanning seven documents [66,73,79-83]. Two CMOCs provide evidence for when a school-based intervention strategy is successful for adolescents in low-resource settings and one for when it is not. This PT explains the importance of creating a supportive environment at the school to promote better learning outcomes, motivate students to continue education and thereby delay adolescent marriage and pregnancy.

This PT describes the importance of providing need-specific, multimedia, problem-based learning education to underperforming girls. It also highlights the involvement of teachers and local mentors in providing after-school support to improve girls’ skills and discuss gender-related topics. Combining these can motivate female students to continue their education and inculcate positive social norm change in the school environment. This intervention can involve providing digital education resources in classrooms, identifying the needs of students and catering to them specifically in after-school sessions, for example. Ainul et al. (2022) explain how their intervention created these supportive spaces:

*Schools were used as the hub of the intervention where girls and families can feel safe and benefit from direct involvement and guidance from trusted and revered teachers who have high status in the community. The constant engagement and support of mentors through weekly meetings with adolescent girls created opportunities to interact, share successes and challenges, exchange ideas and learning, and build adolescent girls’ social networks* [73].

The individual attention and assistance provided to girls will increase their confidence, motivate them to perform better at school, and aspire to roles different from those conventionally assigned to women. This would subsequently lead to girls finishing school, engaging in economic activities and delaying child marriage and pregnancy. However, there are multiple reasons that the intervention may not work, including inadequate training for upskilling teachers, overburdened teachers, and inability to engage in after-school activities.

#### Programme theory 2 – Empowering girls through CSE in safe spaces

If school-going and out-of-school adolescents (C1) living in areas affected by gender inequities (*e.g*. high school dropout of girls, high prevalence of HIV/AIDS, early sexual debut for girls, child or early marriage and teenage pregnancy) (C2) are provided CSE (including HIV/AIDS prevention, gender stereotypes, relative risk messaging, risky sexual behaviours and abstinence education, and in-class quiz or puzzle on these topics) using digital media (M-Res1) in a safe space (M-Res2) by trained facilitators (local female consultants or school teachers) (M-Res3)

Then girls are likely to adopt less risky sexual behaviours such as using contraceptives, sex with fewer partners or similarly aged partners, postponing sex, and abstinence (IO).

Because discussion with trained facilitators and solving quizzes promote critical thinking and help process one's thoughts and beliefs (M-Rea1). Access to information delivered using visual aids increases knowledge retention and knowledge about modern contraceptives, and engagement with these materials helps with goal setting, decision making and planning for suitable outcomes (M-Rea2). Interaction with peers increases trust and develops negotiation skills and conformity to positive health behaviours (M-Rea3). Furthermore, trained facilitators can promote a gender-positive environment in the classroom because they have themselves critically reflected on SRH topics and are sensitised to discussing them (M-Rea4). As a result, both school-going and out-of-school adolescents have more personal and relational control over their sexual health (M-Rea5). They feel supported and connected to their peers and teachers and make informed choices (M-Rea6), which might help prevent adolescent pregnancy (O).

The second PT is supported by six CMOCs extracted from three interventions across seven documents [58,64,66,79,81-83]. Two interventions – Sugar Daddy and Research Initiative to Support the Empowerment of Girls (RISE) – provided evidence for the success of the PT and DREAMS when it might fail. This PT elucidates how interactive CSE delivered in safe spaces by local, female, trained facilitators can empower adolescent girls living in highly gender-disparate areas to adopt less risky sexual health behaviours and, in turn, reduce adolescent pregnancy.

Comprehensive sexuality education components may include engaging adolescent girls in a range of CSE topics such as HIV/AIDS prevention, gender stereotypes, relative risk messaging to choose younger partners in comparison to older partners, risky sexual behaviours and abstinence using digital media material and in-class quizzes. An important feature of this PT involves trained mentors whom the girls can look up to as role models, including teachers or external consultants. As Dupas et al. (2018) [85] describe, ‘students proved responsive to both school staff and external consultants, indicating that teachers do not lack legitimacy or confidence’. Further, this intervention also notes that the use of in-class quizzes/puzzles effectively changed risk perception and beliefs around whom to have sex with and whether to have sex or not. The PT operated by supporting girls to develop a concrete plan concerning their future sexual behaviour, actively adapting their beliefs and behaviour. However, the absence of such activities could result in the failure of the PT. Furthermore, if the local context of the intervention is not gender-disparate or if the adolescents already have the knowledge of health risks, this PT might not be successful.

#### Programme theory 3 – Empowering boys through gender norms-based sexuality and relationship education

If school-going and out-of-school adolescent boys (C1) living in areas affected by gender inequities (*e.g*. high prevalence of HIV/AIDS, teenage pregnancy, and low access to SRH services) (C2) are involved in group-based, context-specific activities like interactive CSE (including television drama showcasing a young couple's journey with adolescent pregnancy, activities to identify gender roles and biases, and exercises to identify trusted adults to discuss SRH topics) (M-Res1).

Then, they are likely to challenge existing beliefs on gender roles and responsibilities, increase the use of contraceptives, delay the initiation of sexual intercourse, and subsequently lower the incidence of adolescent pregnancy (IO).

Because watching an interactive drama with peers would capture attention, evoke empathy, improve their understanding of social influences and norms around masculinity and make the information more relatable (M-Rea1). The gender-role activities would serve as behavioural modelling that would shape participants' perceptions and actions by applying knowledge on maintaining healthy and trusting relationships (M-Rea2). Discussions with peers foster dialogue, peer support, and reflection, potentially leading to shifts in attitudes, norms, and communication patterns within them (M-Rea3). Identifying and interacting with an adult of their choice would give them a sense of agency and spread positive gender norms (M-Rea4). As a result, adolescent boys would be motivated to adopt positive subjective norms, understand its perceived benefits (M-Rea5) and have the confidence to change their sexual behaviour (M-Rea6), which might reduce adolescent pregnancy (O).

This PT is supported by two CMOCs extracted from one intervention that was reported across three documents [59,60,84]. The ongoing project, If I Were Thabo in Lesotho and South Africa, was adapted from If I Were Jack in the UK, which showed the potential to reduce adolescent pregnancy by improving SRH knowledge, positive male gender norms and intentions to avoid unintended pregnancy among adolescents [86]. The ongoing intervention emphasises the importance of norms-based sexual health education specifically targeted towards boys (but also including girls) to support them in understanding the social influences and norms around masculinity, aiming to motivate them to adopt less harmful sexual behaviours.

Activities to engage adolescents include a group-based interactive screening of a television drama showcasing a young couple's journey with adolescent pregnancy, in-class discussions and role plays, and educational materials for parents. As elaborated by Hunt et al.:

*Through engaging in these activities, adolescents are equipped with knowledge, given an opportunity to challenge beliefs and improve their understanding of social influences, and – ultimately – alter their intentions and behaviour in relation to sexual health, including by avoiding unprotected sex* [59].

Further, participating in gender role activities might also serve as a behavioural modelling technique where adolescents can apply the knowledge learnt to maintain healthy and trusting relationships. As a result, they will adopt positive subjective norms and modify their sexual behaviour, which might reduce adolescent pregnancy.

### Programme theories for interventions delivered at the health facility setting for adolescents

#### Programme theory 4 – Confidential youth-friendly health education and services

If school-going adolescent girls and boys (C1) living in areas with high adolescent pregnancy (C2) are provided CSE (M-Res1) in a safe group setting (M-Res2) by trained health providers (M-Res3) along with health services (*e.g*. HIV counselling and testing, information on pregnancy prevention and pregnancy testing, contraceptive counselling, menstrual health and personal hygiene promotion, and hypertension and obesity monitoring) at school via anonymous referral slips or access to services at youth-friendly health facilities outside of school (*e.g*. HIV or sexually transmitted disease prevention, puberty, personal hygiene, menstrual health, and adolescent pregnancy prevention) (M-Res4).

Then, it is likely that their knowledge of sexual health topics and services increases, and they get regular tests and counselling sessions (IO).

Because discussing SRH topics with trained health providers and peers increases knowledge, creates a supportive environment, improves interpersonal relationships with peers, and reduces stigma and hesitation around these topics in a mixed-gender classroom (M-Rea1). Anonymous referral creates autonomy and security for adolescents (M-Rea2), which increases their confidence in the uptake of services, increases demand (M-Rea3) and aids risk reduction (M-Rea4). Access to youth-friendly health facilities removes barriers to accessing care and promotes proactive health-seeking behaviours (M-Rea5).

As a result, adolescents feel supported and safe in accessing health services (M-Rea6) and are motivated to make informed decisions about their sexual health (M-Rea7), which might reduce adolescent pregnancy (O).

The fourth PT is supported by four CMOCs from three interventions spanning three documents [61,68,74]. One intervention Comprehensive sexuality education Health Facility Linkages (CSE-HFL) provides evidence for when the PT might succeed in reducing the incidence of adolescent pregnancy, and two – Economic strengthening and HIV prevention (ES-HIV) and CSE – for when it might fail. It explains that providing CSE in a safe group by trained professionals and access to youth-friendly health services may increase the demand for SRH services. In consequence, adolescents make informed decisions about their sexual health and engage in less risky habits, which results in a decrease in adolescent pregnancy. However, providing CSE without youth-friendly health services and sensitisation of the broader community may lead to failure. This is highlighted by Mbizvo et al., who stated:

*A noteworthy finding of this study was that in-school pregnancies did not decline significantly in the CSE-only control arm, which underscores the role of combining CSE implementation with information on available and responsive SRH services. The effectiveness of CSE implementation may, therefore, hinge upon successful integration with SRH service provision that is adolescent-friendly and attentive to adolescent needs* [61].

Additionally, the confidential use of SRH services by adolescents may be important as parents might disapprove of their use. This was one of the reflections in Wang et al. where they explain:

*This program was controversial, and some parents and two village leaders did not accept it at the beginning, on the assumption that educating adolescents about contraceptives and making contraceptives available would increase the likelihood that young people would engage in sexual intercourse and become pregnant*’ [74].

#### Programme theory 5 – Fostering demand through digital health services and free health supplies

If adolescent girls and boys (C1) living in areas with high prevalence of adolescent pregnancy, premarital sex, HIV/AIDS and sexual violence (C2) are provided with free access to a digital health platform with information on SRH (M-Res1), free access to youth-friendly health facilities with health services (*e.g.* counselling, HIV and pregnancy self-tests, family planning, medicines, contraceptives), and pharmacies selling health products (*e.g.* condoms, emergency contraceptives, oral contraceptive pills, and other medicines) (M-Res2).

Then, they are likely to increase the use of contraceptives, decrease unprotected sex, and increase testing for STIs, which subsequently lead to a reduction in adolescent pregnancy (IO, O).

Because adolescents can access health services and products without financial dependence on parents or partners (M-Rea1). Access to youth-friendly health providers gives non-judgmental care tailored to their unique needs, which will increase trust and support between the health providers and adolescents due to reduced shame and stigma (M-Rea2). They will have control over the type of information they access (M-Rea3), have privacy and anonymity in accessing services and information (M-Rea4), and have decision-making power to plan for suitable outcomes (M-Rea5).

As a result, adolescents feel supported and safe in accessing health services (M-Rea6), are financially empowered to use them (M-Rea7), are motivated to act to improve their sexual and reproductive health and use contraceptives (M-Rea8) which might reduce adolescent pregnancy (O).

The fifth PT is supported by ten CMOCs derived from six interventions reported in 17 documents [57,62,63,65,66,69-72,74,75,77-79,81-83]. Two interventions (one complete – In their Hands (t-safe) programme (ITH) and one ongoing – CyberRwanda) provide evidence of its success, one for its failure (Yathu) and two provide mixed findings (Adolescent Girls Empowerment Programme (AGEP), DREAMS), with some outcomes achieved and others not. The PT describes the utilisation of digital health services, including health apps providing information, games and SRH products via pharmacy, a directory of youth-friendly clinics, loyalty cards, vouchers and free contraceptives, self-test kits, and counselling services.

Providing these health services and products fosters an increase in demand and uptake by adolescents when they are freely available. Hensen et al. support this:

*Adolescents are more likely to be economically dependent than young adults; the reward system likely appeals to them because it provides an opportunity to access products that would otherwise be out of their reach. Providing these products likely encourages adolescents, who may be less likely to consider SRH services important to them, to place greater value on these services* [69].

However, when some health products are not provided freely or when adolescents cannot access them due to mobility barriers, uptake might be hampered, and the PT would fail. Demand can increase when youth-friendly health providers engage in non-judgmental and confidential care tailored to adolescents’ unique needs. However, PT is likely to fail when health providers are not trained in providing sensitive care.

With the availability of a range of digital information, adolescents have increased control over the type of information they access, when, and how to best use it. As the feasibility study by Hemono et al. highlights – ‘students were interested in receiving information about family planning/reproductive health and reported high usage of the webcomic on SRH topics and frequently asked questions (FAQs) and directory of clinics features’ [65]. Like PT2 and PT4, this also promotes decision-making in planning for suitable future health outcomes, such as increasing the use of contraceptives, decreasing unprotected sex, and increasing testing for STIs. However, when service delivery is hampered by depleting stocks or a public health emergency, these positive results would not hold. For example, Hensen et al. explain the potential failure of the intervention due to COVID-19-related restrictions:

*With health hubs closed for three months in response to COVID-19 and stock-outs of oral contraceptives at the hubs and health facilities, it is possible that some youth discontinued use of hormonal contraceptives, particularly the pill, and subsequently lost confidence that services would be offered consistently* [69].

### Programme theories for interventions delivered in the community setting – targeting adolescent girls

#### Programme theory 6 – Empowering girls through employable life skills

If vulnerable adolescent girls, both in-school and out-of-school (C1), living in areas with high gender disparities (*e.g*. low education and sexual knowledge and limited financial resources for girls) (C2) are provided face-to-face structured weekly life-skill sessions (*e.g*. on topics like health, finance, empowerment, computer training and nutrition) (M-Res1) in a safe space with peers (M-Res2), facilitated by trained local female role models /mentors (*e.g*. unmarried, young, educated, skilled women from the community with formal jobs) (M-Res3).

Then, girls are likely to continue their education, engage in various employment activities, and postpone marriage and pregnancy (IO).

Because the life skill sessions equip adolescent girls with practical knowledge and skills like problem-solving, goal setting, decision-making and negotiation, which are essential for their personal and professional development (M-Rea1). The safe space with peers fosters a supportive environment for learning, sharing experiences, and building social connections and trust (M-Rea2). The mentors serve as relatable and aspirational figures for the girls, thus helping girls envision possibilities beyond traditional gender roles, motivating them to aspire to higher education and meaningful employment (M-Rea3).

As a result, girls are empowered (M-Rea4) and have higher human and social capital (M-Rea5), which equips them to overcome gender disparities, pursue employment opportunities, and delay marriage and pregnancy until they are ready (O).

This PT is supported by five CMOCs from four interventions across ten documents [57,63,67,68,71-73,77,78,80]. Here, three interventions support the success of this PT (KGIS, AGEP, Sista), and two explain the failure of the PT (ES-HIV, AGEP). The PT describes the application of in-person life skill sessions facilitated by trained local female role models in a safe space with peers to improve learning outcomes, engage in employment activities, and delay marriage and pregnancies for vulnerable adolescent girls living with gender disparities. This PT enhances girls' practical skills and decision-making capacity, builds social connections, reduces social isolation, and provides aspirational role models. As a result, girls continue their education, engage in employment activities, and postpone marriage and pregnancy. Oberth et al. [67] and Austrian et al. [78] clarify that vulnerable girls who completed all sessions were more likely to observe positive impacts like increased SRH knowledge, contraceptive use, return to school and less likely to become pregnant. However, when participants are unable to attend all sessions, the effect of the intervention disappears. Further, when girls face high resource constraints in attending the skill sessions, the PT also fails. This is verified by Austrian et al. [77], ‘It is also possible that without addressing the economic constraints at the household level, even participation in a girls’ empowerment program alone is not enough to impact longer-term outcomes such as educational attainment or timing of pregnancy’. Building such channels before intervention implementation and ensuring a high participation rate might be helpful in gaining long-term effects.

#### Programme theory 7 – Empowering girls through economic support

If in-school and out-of-school girls from poor families (C1) living in areas with high gender disparities (C2) are provided financial education or economic literacy (M-Res1) and financial assistance in the form of cash transfers, grants to parents or material incentives like payment of school fees (M-Res2).

Then, they are likely to continue schooling, engage in livelihood opportunities, and postpone marriage (IO).

Because girls have decreased financial dependence on families and partners due to cash transfers (M-Rea1), the families have increased financial resources to keep girls in school (M-Rea2). Economic literacy provides girls with essential knowledge and skills to manage their finances effectively, and it empowers them to take control of their economic futures by imparting the skills to advocate for their rights, negotiate for fair wages, access financial services, and pursue entrepreneurial ventures or job opportunities (M-Rea3).

As a result, girls have increased economic and human capital (M-Rea4). They have higher control over available resources (M-Rea5) and decision-making capacity (M-Rea6), which might reduce adolescent pregnancy (O).

The seventh PT is supported by five CMOCs from five interventions (four completed and one ongoing) ranging across 15 documents [57,63,64,66,71-73,76-83]. While two interventions (KGIS, RISE) show when the PT can succeed, three show when it might fail (SIHR, AGEP, DREAMS). The PT highlights how economic support, in the form of financial incentives and financial literacy, can encourage girls from low socioeconomic backgrounds to continue their education, engage in economic activities, and postpone marriage and pregnancy.

It stresses the link between decreased financial dependence on family or partners due to cash transfers or economic skills and increased control over available resources and the decision-making capacity of girls. However, the PT fails when the broader socio-economic context is not conducive to change, *i.e*. there are limited social networks or economic opportunities for girls. Further, when subsidies or cash transfers go directly to the school, the family might not see an overall increase in funds and may not want to invest in their daughters' education. In contrast, Ainul et al. [73] provided direct cash transfers to out-of-school girls to enhance their participation in skill-building activities by counteracting opportunity costs and reducing parental opposition, especially for girls with financial responsibilities and observed a reduction in adolescent pregnancy.

### Programme theories for interventions delivered in the community setting – targeting community members

#### Programme theory 8 – Active involvement and community support for adolescent girls' rights

If stakeholders involved in childcare (including parents, health workers and broader community members) (C1) living in areas with high gender disparities (*e.g*. school dropout, high prevalence of child marriage and adolescent pregnancy) (C2) are engaged in community dialogue (M-Res1) and are provided health information using multimedia channels (radio, TV, in-person activities) on topics related to gender disparities (M-Res2) by trained community mobilisers (*e.g*. mentors, teachers) at a commonplace in the community (M-Res3).

Then, it is likely that school dropout would reduce and child marriage and adolescent pregnancy would decrease (IO, O).

Because there will be an increased understanding of the lived realities of girls in the community (M-Rea1). Community dialogue will promote ownership, buy-in, and commitment to implementing solutions within the community (M-Rea2). Restrictive social norms around these topics will be reduced by fostering a sense of collective responsibility and solidarity in the community (M-Rea3). There will be increased communication and trust between adolescent girls and their caregivers (M-Rea4), and girls will feel supported in voicing their opinions (M-Rea5). Disseminating health information to community members will enhance access to information and services and improve their participation in health-seeking for their children (M-rea6).

As a result, stakeholders strengthen interpersonal and family bonds (M-Rea7). They can better support their children, enabling children to strengthen their self-esteem, develop trust, and find ways to negotiate with caregivers (M-Rea8), which may lead to delay in child marriage, the continuation of school and reduced adolescent pregnancy (O).

This PT is supported by seven CMOCs from seven interventions spanning 16 documents [59-62,64,66,69,70,73,75,79-84]. Six interventions (KGIS, Yathu, CSE-HFL, RISE, ITH) provide evidence for when the PT may succeed and one (DREAMS) for when it might not. It explores the importance of active involvement of the community surrounding adolescents, such as parents, health workers and broader community members, in creating a supportive and gender-equitable environment for adolescents. Various activities conducted by trained community mobilisers, including information on how to access health services, discussing gender disparities and SRH in community forums, and participation of caregivers in school meetings, were applied to promote a dialogue on these topics. This is expected to translate into an increased understanding of the lived realities of girls in the community. At the same time, such interventions may also decrease misinformation on health topics, resulting in reduced adolescent pregnancy. As highlighted by adolescents in interviews by Ajayi et al. [62], ‘the desire to maintain a relationship, poor knowledge of contraceptive methods for preventing unintended pregnancy, misinformation about side effects of modern contraception, and lack of trusted mentors were the main reasons for early pregnancies’. Participants also mentioned that they did not consult anyone because of a lack of trust and the high possibility of breach of confidentiality. This relates to the importance of having a supportive sense of community as proposed by Ainul et al. [73] – a key objective of engaging community members is to create an enabling environment for girls. However, the PT fails for multiple reasons, including if community members and caregivers do not engage in these awareness activities and if the services are of poor quality.

## DISCUSSION

This realist review aimed to synthesise existing evidence on GTIs targeting reduction in adolescent pregnancy in LMICs and to elucidate the mechanisms underlying intervention success or failure. We identified eight programme theories related to three intervention settings – school, health facility and community. Based on the goals of the realist review, we organise our findings as: which interventions work, for whom, how, and under which circumstances.

Multi-component interventions implemented in multiple settings proved more successful in reducing adolescent pregnancy. Specifically, three interventions – KGIS (targeting school and community setting), ITH (targeting health facility and community setting), and CSE-HFL (targeting school, health facility and community setting) – that integrated school, community, and health facility-based activities and resources effectively reduced adolescent pregnancy incidence. However, one intervention, AGEP, despite incorporating community and health facility components, only demonstrated a positive impact among illiterate girls in communities with high premarital-sex prevalence [78]. The limited success of AGEP may be attributed to low levels of participation among girls, alongside insignificant effects on gender-equitable attitudes and safe sex practices, such as delayed sexual debut, contraceptive and condom use, and reduction in multiple sexual partnerships. Although AGEP increased SRH knowledge among participants, the baseline knowledge was very low (1.65 out of 11), and while there was some improvement at the endline (3.67 out of 11), knowledge levels remained low throughout.

Three interventions achieved partial success in reducing adolescent pregnancy. The SIHR intervention (targeting school setting) demonstrated short-term effectiveness, with reduced pregnancy rates two years post-intervention, however, this effect disappeared after five years. In this case, adolescent school girls receiving unconditional cash transfers saw no sustained reduction in adolescent pregnancy, whereas school dropouts receiving conditional cash transfers (CCTs) experienced more reductions. For baseline schoolgirls, CCTs had no impact on fertility. These null results may stem from the intervention’s narrow focus on incentivising schooling without addressing other relevant factors such as health service access, gender norms, SRH knowledge, and sexual behaviour. Second, ‘Sugar Daddy’ (targeting school setting) was successful among girls who dropped out of school and girls in rural areas but did not affect urban school girls [85]. The authors explain that in rural areas, risky sexual behaviour was higher, and HIV knowledge was lower in comparison to urban areas. Presumably, in urban areas, adolescents already had more exposure to information prior to the intervention and experienced lower rates of unwanted pregnancy, and thus experienced smaller impacts [85]. Third, the ‘Sista2Sista’ programme (targeting community setting) showed success only among girls who completed all sessions, indicating that consistent engagement with trained facilitators might be essential for fostering positive attitudes towards contraception and gender-equitable norms. All three of these interventions focused their resources on a single setting, potentially limiting broader or longer-term impacts.

In contrast, four interventions – CSE (targeting community setting), DREAMS (targeting school, health facility and community setting), Economic strengthening and HIV prevention (ES HIV) (targeting school setting), and Yathu Yathu (targeting community and health facility setting) – did not demonstrate a reduction in adolescent pregnancy incidence. For CSE, the intervention included both adolescents and youth in the age group of 15–24 and did not provide age-disaggregated estimates of adolescent pregnancy. Since the mean age of participants in this study was 18.5 years, aggregate estimates may obscure adolescent-specific impacts. In the case of DREAMS, low uptake rates – only 51% attendance for educational sessions and just 1% among caregivers likely hindered its effectiveness [82]. The ES HIV intervention did not have a significant overall effect on adolescent pregnancy; however, absolute numbers over baseline and endline show an increase in pregnancy from 2.3 to 5.6%. This is potentially due to an increase in unprotected sex, transactional sex and the number of sexual partners [68]. Without broader community or family engagement, the intervention’s exclusive focus on adolescents may have limited its capacity for lasting behavioural change. Lastly, for Yathu Yathu, while the intervention had a positive effect on the primary outcome of knowledge of HIV status, it did not affect adolescent pregnancy [69]. This may be related to the intervention’s limited effects on contraceptive use and health care access due to the COVID-19-induced closure of youth hubs and stock-outs of oral contraceptives.

Three interventions (RISE, CyberRwanda, If I Were Thabo) were ongoing. Despite our efforts to locate interventions targeting exclusively boys, we could only find one intervention – If I Were Thabo. While some interventions included boys in their activities, they did not report outcomes on gender norm change, empowerment and fatherhood for boys. This points towards the need to actively involve boys in interventions as a target population and to examine the complex social, cultural and economic dynamics influencing intervention implementation.

In the school setting, PTs aimed at creating a supportive and trusting school climate. The mechanisms of change were related to knowledge retention, uptake of positive subjective norms, critical thinking, fostering communication and trust, and building adolescents’ personal and relational control over sexual health. To this end, these PTs share commonalities with the WHO’s Health Promoting Schools framework, which also highlights the importance of developing healthy school policies that activate many of the above-mentioned mechanisms to promote health and well-being among students [87]. A systematic review of such interventions shows that they can positively affect body mass index, tobacco use, bullying, physical activity, and dietary intake. However, they show no gains in sexual health, including adolescent pregnancy [88]. Similar to the interventions included here, another review based in high-income countries highlighted that school-based interventions can prevent adolescent pregnancy [89]. Future whole-school interventions in LMICs can also explore integrating adolescent pregnancy as an outcome.

The process of change in school-level PTs was supported by local mentors or facilitators in a safe, peer-group-based setting to create opportunities for discussion, exchange and behaviour modelling. School-based peer group activities have been used in other fields, such as violence against women, where they have improved gender-equitable attitudes and norms [90,91]. In many interventions, teachers are also involved in delivering the activities, but their involvement might lead to the failure of the PT when they are not trained adequately, have high workloads or when the hierarchy between the student and teacher is restrictive. This is corroborated by an Indian school-based intervention on school climate and health outcomes, SEHER, where authors found that when the intervention was led by teachers, there was no effect on attitudes towards gender equity and knowledge of SRH topics in comparison to when the intervention was delivered by local lay counsellors [92]. Other studies from high income countries have also shown that building a sense of community in schools and providing a safe space for students to interact can lead to better health outcomes [93,94].

The three PTs at the school level can be traced to higher-order theories like learning, empowerment and behaviour change theory. Learning and behaviour change theories help us understand how individuals acquire knowledge, skills, and behaviours through various learning processes and how to optimise them to facilitate desired outcomes. A classroom or peer group is where socialisation in a wider community occurs, along with identity and norm formation, which is important to understand one's reflections and those of others [95-98]. This interaction helps form social capital among adolescents, which translates into empowerment. As conceptualised by Kabeer, empowerment theory includes assets and opportunities as resources and agency, which is the capacity of action to turn these resources into achievable goals or outcomes [99]. This forms the core of GTIs, which aim to induce resources that were previously not available to adolescents and communities to trigger aspirations and capabilities in them to achieve the goal of preventing adolescent pregnancy. However, where most interventions fail is assuming that their activities will positively affect norms and empowerment but fail to measure or report changes in them.

Programme theories in the health facility setting aim to create supportive, youth-friendly and accessible health environments that give adolescents control over how and where they access SRH information. Other youth-friendly digital health interventions for SRH in LMICs have also shown similar results in changing attitudes and reducing misconceptions about contraceptive use [100,101]. Recent reviews also highlight that trained providers, respect and privacy, confidentiality, and quality services are the main facilitators of the uptake of SRH services among adolescents [102,103].

The PT developed at the health facility setting can be mapped onto empowerment, social norms and behaviour change theories. A recent framework, Evidence-based Measures of Empowerment for Research on Gender Equality (EMERGE), conceptualises these theories to measure empowerment for health and development [104]. Like the PTs mentioned above, the EMERGE framework highlights that the activation of mechanisms happens in the broader context of a supportive external environment with positive internal norms and beliefs. However, access to services and information in a supportive environment will not be enough on its own. Rather, the translation of norms into positive health behaviour will be the core [105]. In the PTs, this happens as schools and health facilities become the external environments that promote self-actualisation and goal-setting among adolescents. The inclusion of youth-friendly practices and peer group activities influences and promotes change in health behaviour through social interaction, thereby leading to desire, motivation and conviction to achieve the self-determined goal of pregnancy prevention.

Finally, in the community setting, PTs targeted both adolescent girls and the communities they lived in, which were often resource-poor and had high gender disparities. Here, PTs often involved role models in conducting training sessions to foster a sense of community and support, as in PT 1, and to activate mechanisms of motivation and aspiration for girls to envision possibilities beyond traditional gender roles. Mentoring and role model-based interventions in science, technology, engineering, and mathematics (STEM) and entrepreneurship research have positively affected female students’ attitudes towards science and technology issues, improved opinions of related vocations, increased entrepreneurial intentions, reduced gender stereotypes and increased self-employment [106-108]. Discussing SRH topics with mentors has also been shown to improve outcomes, such as knowledge, childbearing age, violence experience, and increased social networks [109,110]. Increased demand for health services and acceptance in the community are linked to the supply-side strategies of youth-friendly services. Previous studies have highlighted that involving key community gatekeepers such as parents and religious leaders can create wider support [111,112]. Other studies on parent-child communication have also been shown to increase pregnancy knowledge and access to contraceptives [113,114]. This review did not find any interventions solely focusing on the household setting. Future studies could explore developing and implementing interventions within the family setting.

These PTs focused on mechanisms to build human, social and economic capital among adolescent girls. These mechanisms fall under Pierre Bourdieu's and James Coleman’s social capital theories and Kabeer’s empowerment theory by enhancing community bonds and networks where girls can gain agency and resources to achieve their health goals [99,115]. Many economic empowerment interventions also target these mechanisms to address child marriage, HIV and gender-based violence [52,116-118]. Like the PTs developed here, other qualitative studies have highlighted that economic incentives promote a sense of financial independence, adult social identity and hope for a better future among girls, leading to a reduction in transactional sex and risky sexual behaviours [117,119].

Our findings show that interventions that place their inputs in multiple settings (*e.g*. CSE-HFL, KGIS, ITH) are better suited to reducing adolescent pregnancy. This integration is important to bring about the effective translation of knowledge to the practice of healthy behaviours, as highlighted by Akinwale et al. [120]. Their systematic review underscores that most of the included studies reported low levels of adolescent awareness of SRH topics due to insufficient information about where and how to access these services. Linking CSE programmes at schools with health services is a good way to tackle this barrier, as shown in this review. However, the PTs may fail when the interventions do not tackle structural and implementation pitfalls, including long distances to obtain services, financial constraints in reaching and obtaining services and lack of support within the family, amongst other barriers [111,120,121]. Integrated interventions spanning school, health facilities, and community settings may hold the greatest potential for success in reducing adolescent pregnancy by providing a more conducive environment for empowerment and behaviour change than isolated interventions. Integration ensures that SRH knowledge gained at school is supported by access to youth-friendly health services and reinforced through positive community and family norms. This alignment across settings helps address the socio-cultural, structural, and interpersonal factors that affect adolescents' health decisions and behaviours. The conceptual framework proposed in this review highlights the importance of a supportive continuum between these settings.

This review’s main strength is the realist approach, which focuses on unravelling the potential intervention mechanisms. In the supplement, we provide a transparent record of all the processes followed and how decisions at each stage were made. The inclusion of experts at various stages of theory formulation also adds to a critical review of proposed theories and their relevance to the field of SRH. While few studies in this review included a clearly stated theory of change underlying the intervention, we utilised the established theories along with the realist analysis to outline eight testable causal explanations for outcomes, detailing how they work, for whom and under which circumstances. The review has limitations as well. First, our search was limited to English-language articles. Further, in this review, the majority of the interventions were located in SSA. Future studies in other regions could explore integrating adolescent pregnancy as an outcome within their SRH outcomes. Second, we only included interventions that were explicitly gender transformative, as outlined in the previously published protocol. This prevented us from including insights from structural interventions like country-level poverty alleviation programmes or education programmes that also had an impact on adolescent pregnancy, like the Kenya Cash Transfer [122]. Third, we only included interventions that measured the incidence of adolescent pregnancy, leading to the exclusion of interventions that measure changes in knowledge and attitudes or only contraceptive use as an outcome. Fourth, even after actively searching for qualitative insights from the interventions, we could locate few, mainly because they might not always be recorded or published. Future studies could consider publishing supplementary material, including the complete set of variables measured, pilot studies, challenges to intervention implementation and assumptions on the theory of change. Considering the implementation process is equally important here. Future interventions could consider applying frameworks like Context And Implementation Of Complex Interventions (CICI), Medical Research Council (MRC) and Template For Intervention Description And Replication (TIDieR) to describe intervention characteristics, reflect on context, and assess feasibility and acceptability [123-125]. Lastly, the PTs presented here are not causally definitive. Based on the evidence, we believe that these mechanisms are necessary for reducing adolescent pregnancy. However, this requires further empirical testing. Future studies could explore testing these PTs for further causal explanations.

## CONCLUSIONS

This realist review developed eight programme theories to understand how GTIs work, for whom and under which circumstances. The evidence suggests that GTIs are most successful when they are multi-component and target adolescents facing high gender inequalities like school dropout, high prevalence of HIV or violence, early sexual debut, child or early marriage, low access to SRH services and limited financial resources. However, targeting the most vulnerable adolescents can also backfire if interventions do not consider the structural context. Therefore, future GTIs should consider the local conditions that would help them succeed, for example, noting the main SRH policies in the country, socio-economic and employment characteristics of the region, availability of health facilities and schools, and household responsibilities of participants that could act as a barrier to participation. To maximise their potential, GTIs should have a clear focus, indicate the pathways of influence and assumptions underlying them and assess how they work to reduce restrictive social norms and increase empowerment by using appropriate indicators.

## Additional material


Online Supplementary Document
