# Supplementary material for: How do gender transformative interventions reduce adolescent pregnancy in low- and middle-income countries: a realist synthesis
Source: J Glob Health. 2025 Apr 4;15:04102. doi: 10.7189/jogh.15.04102 (PMC11969290; doi:10.7189/jogh.15.04102)
Supplement: Online Supplementary Document [file jogh-15-04102-s001.pdf]

Supplement to: Shukla S, Kharade A, Böhret I, Jumaniyazova M, Omolade Abejirinde IO, Meyer SR, Shenderovich Y, Steinert JI. How do gender transformative interventions reduce adolescent pregnancy in low and middle-income countries? A realist synthesis. J Glob Health. 2025;15:04102.

Table of Contents

Document 1: List of studies used to develop the IPTs 2

Document 2: CMO extraction codebook 1 3

Document 3: Initial intervention framework 4

Document 4: Initial programme theories 5

Document 5: Expert Consultation questions (Round 1 – IPT) 6

Document 6: Search Strategy 7

Document 7: Appraisal tool - Relevance, Richness, Rigour 8

Document 8: Data extraction form 9

Document 9: Feedback form for experts on refined theories.... 10

Document 10: RAMESES publication standards for realist synthesis.... 11

Document 11: CMOCs table 14

Document 12: Programme theories and associated CMOCs 79

Document 1: List of studies used to develop the IPTs

Alam 2010:<br>Alam, Andaleeb, Baez, Javier E., and Del Carpio, Ximena V. Does Cash for School Influence Young Women's Behavior in the Longer Term? Evidence from Pakistan. Policy Research Working Papers. May 2011.
Angrist 2019:<br>Angrist, N., Matshaba, M., Gabaitiri, L. et al. Revealing a safer sex option to reduce HIV risk: a cluster-randomized trial in Botswana. BMC Public Health 19, 610 (2019). https://doi.org/10.1186/s12889-019-6844-8
Austrian 2021:<br>Austrian, K., Soler-Hampejsek, E., Kangwana, B. et al. Impacts of two-year multisectoral cash plus programs on young adolescent girls' education, health and economic outcomes: Adolescent Girls Initiative-Kenya (AGI-K) randomized trial. BMC Public Health 21, 2159 (2021). https://doi.org/10.1186/s12889-021-12224-3
Austrian 2022:<br>Austrian, K., Soler-Hampejsek, E., Kangwana, B., Maddox, N., Diaw, M., Wado, Y. D., ... & Maluccio, J. A. (2022). Impacts of multisectoral cash plus programs on marriage and fertility after 4 years in pastoralist Kenya: a randomized trial. Journal of Adolescent Health, 70(6), 885-894.
Kangwana 2022:<br>Koech, J., Maluccio, J. A., Kangwana, B., Austrian, K., Soler-Hampejsek, E., Maddox, N., ... & Mbushi, F. (2022). Impacts of multisectoral cash plus programs after four years in an urban informal settlement: Adolescent Girls Initiative–Kenya (AGI–K) randomized trial.
Baird 2010:<br>Baird, S., Chirwa, E., McIntosh, C., & Özler, B. (2010). The short-term impacts of a schooling conditional cash transfer program on the sexual behavior of young women. Health economics, 19(S1), 55-68.
Baird Report 2010:<br>Baird, Sarah and McIntosh, Craig and Ozler, Berk, Cash or Condition? Evidence from a Cash Transfer Experiment (March 1, 2010). World Bank Policy Research Working Paper No. 5259, Available at SSRN: https://ssrn.com/abstract=1585038
Baird 2015:<br>Baird, S., Chirwa, E., McIntosh, C., & Özler, B. (2015). What happens once the intervention ends? The medium-term impacts of a cash transfer programme in Malawi. Impact Evaluation Report, 27.
Bandiera 2012:<br>Bandiera, O., Buehren, N., Burgess, R., Goldstein, M., Gulesci, S., Rasul, I., & Sulaiman, M. (2012). Empowering adolescent girls: evidence from a randomized control trial in Uganda (pp. 1-13). Washington, DC: World Bank.
Buchmann 2017:<br>Buchmann, N., Field, E., Glennerster, R., Nazneen, S., Pimkina, S., & Sen, I. (2017). Power vs money: Alternative approaches to reducing child marriage in Bangladesh, a randomized control trial. Unpublished Manuscript.
Duflo 2015:<br>Duflo, E., Dupas, P., & Kremer, M. (2015). Education, HIV, and early fertility: Experimental evidence from Kenya. American Economic Review, 105(9), 2757-2797.
Dunbar 2014:<br>Dunbar, M. S., Kang Dufour, M. S., Lambdin, B., Mudekunye-Mahaka, I., Nhamo, D., & Padian, N. S. (2014). The SHAZ! project: results from a pilot randomized trial of a structural intervention to prevent HIV among adolescent women in Zimbabwe. PloS one, 9(11), e113621.
Dupas 2011:<br>Dupas, P. (2011). Do teenagers respond to HIV risk information? Evidence from a field experiment in Kenya. American Economic Journal: Applied Economics, 3(1), 1-34.
Dupas 2018:<br>Dupas, P., Huillery, E., & Seban, J. (2018). Risk information, risk salience, and adolescent sexual behavior: Experimental evidence from Cameroon. Journal of Economic Behavior & Organization, 145, 151-175.
Ross 2007:<br>Ross, D. A., Changalucha, J., Obasi, A. I., Todd, J., Plummer, M. L., Cleophas-Mazige, B., ... & Hayes, R. J. (2007). Biological and behavioural impact of an adolescent sexual health intervention in Tanzania: a community-randomized trial. Aids, 21(14), 1943-1955.
Cowan, 2010:<br>Cowan, F. M., Pascoe, S. J., Langhaug, L. F., Mavhu, W., Chidiya, S., Jaffar, S., ... & Regai Dzive Shiri Trial Team. (2010). The Regai Dzive Shiri project: results of a randomized trial of an HIV prevention intervention for youth. Aids, 24(16), 2541-2552.
Jewkes 2008:<br>Jewkes, R., Nduna, M., Levin, J., Jama, N., Dunkle, K., Puren, A., & Duvvury, N. (2008). Impact of stepping stones on incidence of HIV and HSV-2 and sexual behaviour in rural South Africa: cluster randomised controlled trial. Bmj, 337. https://doi.org/10.1136/bmj.a506
Mehra 2018:<br>Mehra, D., Sarkar, A., Sreenath, P. et al. Effectiveness of a community based intervention to delay early marriage, early pregnancy and improve school retention among adolescents in India. BMC Public Health 18, 732 (2018). https://doi.org/10.1186/s12889-018-5586-3

Document 2: CMO extraction codebook 1

Study details										
Study ID	Publication type	Publication year	Geographic area	Target population	Target Age	Sampling	Sample characteristics	Sample size (only adolescents)	Type of study	Aim of study

Intervention						
Intervention name	Aim	Intervention Strategy (e.g. education, skills training, communication campaign etc.)	Details/ Activities/ Content	Duration/ Frequency	Adaptation (from another intervention)	Description of staff, their training, their supervision

Context		Mechanism
Setting (i.e. community, school etc; region characteristics)	Individual characteristics (participant's family/HH background, economic back. etc)	Theoretical or hypothesised in the intro/ methods/ discussion/ results)

Outcome			
Type of outcomes	Measures (Outcome of interest)	Time frame of measures	Findings (including adverse effects)

Document 3: Initial intervention framework

Context

Individual
Age groups, unmarried, in-school, school drop-out, knowledge on HIV transmission, transactional sex

Family
Multigenerational households, socioeconomic background, poverty, poor housing

School
Unable to pay school fees, poor school facilities, inadequate number of schools, high rates of drop-out, low enrollment of girls

Community/Society
Minority groups, infrastructure, restricted mobility, low SRH services, high migration, low literacy, high HIV prevalence, restrictive gender and social norms, high fertility rate, pregnancy outside/within marriage

Policy Environment
Poor government services, civil unrest, climate change, no condom distribution policy, education sector reform

Intervention Strategies

Economic strengthening
Conditional cash transfer (on school attendance), unconditional cash transfer, in-kind gifts like cooking oil, school uniform, school kits

Financial education
Savings skills training and financial education, microfinance and credit, vocational training on small-scale business

Life-skills training
Management skills, negotiation and conflict resolution, leadership, labour market participation and oral communication

Health education
HIV/AIDS education (pre-natal and post-natal), comprehensive sexual health education (menstruation and menstrual disorders, pregnancy, sexually transmitted infections, family planning, violence prevention, healthy relationships)

Service provision
Healthcare services like contraceptive provision, mental health, guidance counselling

Gender/Social norm change
Role models or mentors, popular culture, policy reform

Note: the intervention strategies can be used in combination with each other.

Implementation components

Location: school, community club, youth club, adolescent club, safe spaces, health club at school, youth information centre, clubs at multiple locations, online spaces

Intervention provider: same-sex trained mentors, teachers, facilitators, trainers, entrepreneurs, consultants, project officers, youth trainers, experienced, from same communities, health workers, government workers, religious leaders

Procedure and Material: region and context-specific quizzes, discussions, movies, puzzles, ice breakers, lectures, posters, games, picture cards, campaigns, peer condom social marketing, helpline, crisis centres, sports, peer learning, digital resources on SRH

Fidelity: Monitoring visits, quality checks, surprise visits, training ranging from 4 to 14 days, training curriculum, monthly meetings, protocols and checklists, refresher training, multisector collaboration, comparison group, duration of activities, participation rate

Fidelity assessment: Process evaluations, cost-benefit analysis

Modification/Tailoring: Pilot, adapted from another context, small scale, large scale, personalised

Mechanism

Empowerment: resources, agency, decision-making capacity, achievement of capabilities
Economic empowerment: access to funds, control over funds and benefit from them, power to make decision
Learning/experiential learning: experience, retention of knowledge/skills, reflection, reproduction of skills, motivation to do more
Social capital: trust, feeling of belonging, participation, relationship with others, acceptance/approval from others
Human capital: knowledge, skill, judgement
Self-Determination: autonomy, competence, relatedness to others
Peer relations: social comparison, conformity, modelling, peer pressure, socialization and feedback
Collective efficacy: shared experiences, emotional support, vicarious learning and role modelling

Outcome

Intermediate: contraceptive use, delay in sexual debut, delay in early marriage, continued schooling, changes in gender and social norms, decreased household economic stress, access to SRH services

Distal: Reduced adolescent childbearing or unwanted or unintended pregnancy/ decrease in the number of births to under 19 girls/ increase in adolescent pregnancy/ delayed age at first birth/ reduction in adolescent fertility rate/ reduction in repeat adolescent pregnancy

Document 4: Initial programme theories

Initial programme theories

1. If adolescent girls living in low socioeconomic areas with limited economic resources (C) receive free education and financial assistance to attend school, mainly secondary and higher secondary level (M-Resource), then they tend to marry later in life and have fewer children (O) because education empowers them to decide independently and gives them the confidence to voice their opinions (M-Reasoning). Further, economic assistance increases the family's income (M-Resource), enabling them to sustain the girl's education without compromising on essential expenses (M-Reasoning).

2. If adolescent girls residing in HIV-prevalent areas (C) are provided comprehensive sex education in junior school, facilitated by local, trained, and motivated educators of the same sex, utilising interactive techniques such as videos, quizzes, problem-solving games, and group discussions (M-Resource), then her age of sexual debut increases, resulting in delayed initiation of sexual activity and the number of sexual partners she engages with also decreases, leading to a reduction in risky sexual behaviour (O) because she has accurate and reliable information about safe sex practices to make responsible choices (M-Reasoning) and because her beliefs about gender roles change promoting more equitable attitudes and behaviours (M-Reasoning).

3. If adolescent girls residing in conflict-prone areas with prevalent restrictive social norms such as child marriage and bride price (C) are exposed to intervention activities aimed at reducing gender-based violence, providing educational supplies and cash transfers, offering health education, and facilitating money-saving activities through trained local mentors (M-Resource), then girls use condoms and delay sexual debut which contributes to a decline in overall rates of adolescent pregnancy (O) because they can negotiate for a safer sexual environment, assert their boundaries and advocate for their sexual health (M-Reasoning). They are motivated to set goals and apply the knowledge they acquire to generate funds and improve their economic situation because their decision-making capacity improves in favour of long-term goals and well-being (M-Reasoning).

4. If out-of-school adolescent girls living in densely populated communities (C) are provided with health services, sex education, vocational training, micro-credit, and social support services coupled with counselling (M-Resource), then rates of childbearing reduce (O) because girls have access to income-generating opportunities which increases their financial independence and mobility (M-Reasoning). They have access to health services that enhance their ability to make choices that align with their desired outcomes, and they have greater agency and control over their lives through the guidance and support of counsellors (M-Reasoning).

5. If adolescent girls living in rural communities with a high prevalence of child marriage and low socioeconomic status (C) are offered a safe space facilitated by trained, local same-sex mentors to openly discuss sexual and reproductive health and rights, in addition to receiving soft-skills training (M-Resource), then adolescent pregnancy reduces (O) because adolescent girls gain a sense of belonging and support, enabling them to navigate challenges related to sexual and reproductive health, they gain valuable abilities such as communication, negotiation, and critical thinking to understand their rights better, communicate their needs effectively, and make informed choices regarding their sexual and reproductive health (M-Reasoning). Further, they engage in dialogue with peers and challenge and reshape traditional beliefs and practices around child marriage and girls' household decision-making power (M- Reasoning).

6. If young boys and girls living in rural settings (C) are provided with an integrated intervention with a focus on effective communication within the community, training health workers to deliver youth-friendly services, and ensuring access to comprehensive health services (M-Resource), then adolescent pregnancy reduces (O) because gendered beliefs among community members and within families undergo a positive transformation as communication helps break down barriers and stigmas surrounding reproductive health (M-Reasoning). Further, individuals gain knowledge on reproductive health issues, including pregnancy prevention and family planning (M-Resources), and this newfound understanding empowers them to challenge and modify their existing gendered beliefs (M-Reasoning). Finally, accessible and youth-friendly healthcare (M-Resources) empowers individuals to seek assistance and make decisions that align with their reproductive goals (M-Reasoning).

7. If adolescent girls aged 14-20 years living in communities with restrictive gender norms (C) are provided with an integrated intervention at an adolescent club in the community, including vocational skills training like hairdressing, tailoring, computing, agriculture, and small trades; life skills education on negotiation, conflict resolution, and leadership; sexual and reproductive health education; mentorship; and microfinance facilitated by trained female mentors from their community (M-Resources) then condom use increases and incidence of childbearing reduces (O) because girls engage in income generating activities thereby improving their economic prospects and financial independence and reduce dependency on men (M-Reasoning). Further, girls' knowledge of health-related topics (M-Resources) increases their self-confidence and decision-making skills, enabling them to make informed choices regarding their health, relationships, and future aspirations (M-Reasoning). Lastly, clubs provide a supportive and safe environment (M-Resources) and enable girls to form close connections with their peers, thereby increasing their social capital (M-Reasoning).

8. If adolescent girls at school (C) are provided HIV-related sex education on the risks associated with relationships with older men using in-class quizzes, discussions, videos, and lectures by trained teachers (M-Resources), then unprotected sex and teenage pregnancy will reduce (O) because girls will engage in concrete plan formation about reducing their relative risk of pregnancy with older vs younger partners by planning for specific strategies, such as abstaining from sexual activity (M-Reasoning). Further, knowledge about HIV transmission and prevention (M-Resources) will enhance their understanding of applying different strategies. Lastly, observing their peer's reactions in a group setting (M-Resources) will influence girls' second-order beliefs, leading them to align their behaviours with what they perceive as socially desirable and safe (M-Reasoning).

Document 5: Expert Consultation questions (Round 1 – IPT)

Based on your experience in the implementation of science/ sexual and reproductive health programs:

1. Are the context categories mentioned in the initial program theory relevant to gender transformative interventions? What else would you add to these categories that we still need to consider? Or What would you delete, given they are not as important? Or would you suggest a different set of categories?
2. What do you think about the intervention strategies and techniques mentioned in the flowchart? Are they well suited to impact adolescent pregnancy? When will they make the maximum impact? Will you add another strategy/technique or delete any of them, why?
3. What is your opinion on how a gender transformative intervention might cause its outcomes? How do you think the mentioned IPTs might cause or help to cause a reduction in adolescent pregnancy?
4. What do you think about the proposed mechanisms in the IPTs? Which mechanisms, according to you, are the most important in bringing long-lasting behaviour change?
5. Do you think the outcomes will be the same for all adolescents (boys, girls)? In what ways will they be different?
6. If you could change something about the IPTs to make it more relevant or understandable, what would you change and why?

Document 6: Search Strategy

Number	Criteria	Terms
1	Adolescent	adolescent* OR teen* OR young people OR youth* OR school age* OR juvenile* OR minor OR minors OR youngster* OR underage* OR teenager* OR emerging adult* OR early adulthood OR young adult* OR young women OR young men OR boy OR boys OR girl OR girls
2	Adolescent pregnancy	Adolescent pregnancy OR teen pregnancy OR teenage pregnancy OR young maternal age OR early pregnancy OR unintended pregnancy OR unwanted pregnancy OR adolescent childbearing OR adolescent motherhood OR teenage motherhood OR teenage childbearing OR young maternal health OR adolescent fertility
3	Gender transformative interventions	GTP OR GTI OR gender transformative interventions OR gender transformative programs OR gender transformative approaches OR GTA OR gender transformative initiative OR gender program OR gender intervention OR gender project OR gender inequality OR gender norms OR gender OR gender club OR gender training OR power inequity
4	Low- and middle-income countries (LMICs)	Afghanistan OR Albania OR Algeria OR Angola OR Antigua OR Barbuda OR Argentina OR Armenia OR Armenian OR Aruba OR Azerbaijan OR Bahrain OR Bangladesh OR Barbados OR Benin OR Byelarus OR Byelorussian OR Belarus OR Belorussian OR Belorussia OR Belize OR Bhutan OR Bolivia OR Bosnia OR Herzegovina OR Hercegovina OR Botswana OR Brasil OR Brazil of Bulgaria OR Burkina Faso OR Burkina Fasso OR Upper Volta OR Burundi OR Cambodia OR Khmer Republic OR Kampuchea OR Cameroon* OR Cameron OR Camerons OR Cape Verde OR Central African Republic OR Chad OR Chile OR China OR Colombia OR Comoros OR Comoro Islands OR ComORes OR Mayotte OR Congo OR Zaire OR Costa Rica OR Cote dIvoire OR Ivory Coast OR Croatia OR Cuba OR Cyprus OR Czechoslovakia OR Czech Republic OR Slovakia OR Slovak Republic OR Djibouti OR French Somaliland OR Dominica OR Dominican Republic OR East Timor OR East Timur OR Timor Leste OR Ecuador OR Egypt OR United Arab Republic OR El Salvador OR Eritrea OR Estonia OR Ethiopia OR Fiji OR Gabon OR Gabonese Republic OR Gambia OR Gaza OR Georgia Republic OR Georgian Republic OR Ghana OR Gold Coast OR Greece OR Grenada OR Guatemala OR Guinea OR Guam OR Guiana OR Guyana OR Haiti OR Honduras OR Hungary OR India OR Maldives OR Indonesia OR Iran OR Iraq OR Isle of Man OR Jamaica OR Jordan OR Kazhakstan OR Kazakh OR Kenya OR Kiribati OR Korea OR Kosovo OR Kyrgystan OR Kirghizia OR Kyrgyz Republic OR Kirghiz OR Kirgizstan OR Lao PDR OR Laos OR Latvia OR Lebanon OR Lesotho OR Batusoland OR Liberia OR Libya OR Lithuania OR Macedonia OR Madagascar OR Malagasy Republic OR Malaysia OR Malaya OR Malay OR Sabah OR Sarawak OR Malawi OR Nyasaland OR Mali OR Malta OR Marshall Islands OR Mauritania OR Mauritius OR Agalega Islands OR Mexico OR Micronesia OR Middle East OR Moldova OR Moldovia OR Moldovian OR Mongolia OR Montenegro OR Morocco OR Ifni OR Mozambique OR Muanmar OR Myanma OR Burma OR Namibia OR Nepal OR Netherlands Antilles OR New Caledonia OR Nicaragua OR Niger OR Nigeria OR Northern Mariana Islands OR Oman OR Muscat OR Pakistan OR Palau OR Palestine of Panama OR Paraguay OR Peru OR Philippines OR Philipines OR Phillipines OR Phillippines OR Poland OR Portugal OR Puerto Rico OR Romania OR Rumania OR Roumania OR Russia OR Russian OR Rwanda OR Ruanda OR Saint Kitts OR St Kitts OR Nevis OR Saint Lucia OR St Lucia OR Saint Vincent OR St Vincent OR Grenadines OR Samoa OR Samoan Islands OR Navigator Island OR Navigator Islands OR Sao Tome OR Saudi Arabia OR Senegal OR Serbia OR Montenegro OR Seychelles OR Sierra Leone OR Slovenia OR Sri Lanka OR Ceylon OR Solomon Islands OR Somalia OR South Africa OR Sudan OR Suriname OR Surinam OR Swaziland OR Samoa OR Syria OR Tajikistan OR Tadzhikistan OR Tadjikistan OR Tadzhik OR Tanzania OR Thailand OR Togo OR Togolese Republic OR Tonga OR Trinidad OR Tobago OR Tunisia OR Turkey OR Turkmenistan OR Turkmen OR Uganda OR Ukraine OR Uruguay OR USSR OR Soviet Union OR Union of Soviet Socialist Republics OR Uzbekistan OR Uzbek OR Vanuatu OR New Hebrides OR Venezuela OR Vietnam OR Viet Nam OR West Bank OR Yemen OR Yugoslavia OR Zambia OR Zimbabwe OR Rhodesia OR developing countr* OR less* developed countr* OR under developed countr* OR underdeveloped countr* OR middle income countr* OR low* income countr* lmic OR lmics OR low income countr* OR middle income countr* OR low and middle income countr*

Document 7: Appraisal tool - Relevance, Richness, Rigour

Relevance	Richness	Rigour
**Include**<br>1. Documents where the majority of the study participants under the age of 20 OR data is age disaggregated for adolescents, AND<br>2. One of the outcomes is adolescent pregnancy/ adolescent fertility/ age at pregnancy, AND<br>3. Documents from LMICs, AND<br>4. Documents discussing a gender transformative intervention, AND<br>5. Any study design/article type, except for reviews/meta-analyses<br><br>**Exclude**<br>1. Documents with no adolescent in the participant sample, OR<br>2. Documents that do not include adolescent pregnancy as an outcome, OR<br>3. Documents from high-income countries OR<br>4. Documents not including a gender transformative intervention, OR<br>5. Documents that lack full text, systematic review and other types of reviews, meta-analysis	Information on causal pathways, theoretical models, conceptual framework or theory of change involved in the intervention design that explains how it is expected to work.<br><br>Rating:<br><br>1. Low<br>There is little or no information on the intervention context, strategy, implementation process, or mechanism that could contribute to the development of new program theories or refinement of IPTs.<br><br>2. Medium<br>There is some information on intervention context, strategy, implementation process, or mechanism.<br><br>3. High<br>There is a rich description of all aspects of the IPTs, including intervention context, strategy, implementation process, or mechanism.	Trustworthiness and credibility of the data source and methods used for analysis.<br><br>Based on JBL checklists[46], the following questions were devised to evaluate rigour:<br><br>1. The sample size and sampling strategy were adequate<br>2. Data collection and analysis methods were adequate<br>3. Outcome measures were reliable<br>4. Research ethics were followed<br>5. The CMOs listed were justifiable<br><br>Rating:<br><br>1. Low<br>Scores 1 out of 5 measures listed above<br><br>2. Medium<br>Scores 2 out of 5 measures listed above<br><br>3. High<br>Scores 3 or more out of the measures listed above

Document 8: Data extraction form

	Study Details			Study Details									
Extracted by	Study ID	Author names	Paper title	Journal name/source	Publication type	Publication year	Continent	Country	Target population (This can be in combination with adolescents)	Age group of target population included in the study	Adolescent sample size (How many adolescents were included in the study)	Type of study	Aim of study (Paste from paper if available, otherwise write a aim based on own understanding)

Intervention									
Intervention name	Intervention Strategy (e.g. education, skills training, communication campaign etc.)	Details/ Activities/ Content (Write/paste details of each intervention strategy)	Location of intervention (Venue at which the intervention was implemented, eg. class, community, youth club etc)	Intervention target (girls only, girls and parents, girls and boys, etc.)	Duration/ Frequency (How long did the intervention last, how many times was it conducted)	Adaptation (Name and location of the previous intervention)	Description of implementors/staff, their training, their supervision	Monitoring and evaluation	Quality (What methods they used to ensure the quality of the intervention eg. process evaluation, surprise visits etc)

Context		
Setting (Details on community, school, region, country, policy context or characteristics)"	Individual characteristics (Characteristics of adolescents included in the study eg. vulnerability characteristics like ultra poor, orphans, school dropouts etc)	Level of context targeted by intervention (Individual, family, community, school, policy)

Mechanism	
Theoretical or hypothesised (Models/Theoretical Frameworks mentioned in the intro/ methods/ discussion/ results), eg socioecological theory)	Resource and reasoning (Resources: components that are introduced by the programme under study (eg. interaction between students and counsellors). Reasoning refers to human responses triggered by the introduction of the resource. (eg. trust, rapport building)

Outcome			
Measure of adolescent pregnancy	Other outcomes measured	Time frame of measures (Months, time frame per intervention/arm)	Findings (Include adverse effects, impact of the intervention on adolescent pregnancy or other SRH outcomes like condom use, sexual violence etc if it is related to it)

Measures of gender norms or social norms change	Measures of empowerment	Relevant IPT(s)	Additional Information & Excerpts from Text	Notes and Gaps	Cost effectiveness/ Funding	Partners
Details of the variable or outcomes specific to gender norm and social norm change.	Note empowerment outcomes if measured.	Mention which IPT might fit this paper (Refer to the protocol)	Paste details from paper that gives evidence on potential connection between context mechanism and outcome	Notes on decision-making process that you might want to highlight and potential gaps in the study	Details on cost-effectiveness and funding if provided	

Document 9: Feedback form for experts on refined theories

Option 1: On Google forms

Option 2: Feedback after presentation

Option 3: In-person meeting

Introduction: This form/presentation presents eight programme theories (PT) developed by synthesising data during the realist review process on how gender transformative interventions work to prevent adolescent pregnancy in low- and middle-income settings. Kindly provide your feedback on the PT based on your experience in the field of implementation science/sexual and reproductive health programmes/ gender transformative programmes.

For each programme theory:

1. Is the programme theory clear? Does it need more explanation?

- Yes, it is clear
- No, it is unclear
- If unclear: please explain

2. In your experience, would you say that this PT is relevant to gender transformative interventions on adolescent pregnancy?

- Yes
- No
- Other: please explain

3. In your work, have you experienced this PT (strategies and theoretical underpinnings) or forms of this PT?

- Yes, I have seen or experienced this PT
- No, I have not seen this PT but it seems plausible
- No, this differs from my experience
- Other: please explain

4. We found evidence both for and against this PT in how it potentially leads to a reduction in the incidence of adolescent pregnancy. In your expert opinion, could you suggest potential reasons on why this PT might not work to reduce the incidence of adolescent pregnancy?

Open answer:

5. Based on your expertise, do you have any suggestions for refining or strengthening the PT to enhance its explanatory power or practical utility? Any other thoughts on this PT?

Open answer:

Document 10: RAMESES publication standards for realist synthesis

Wong G, Greenhalgh T, Westhorp G, Buckingham J, Pawson R. RAMESES publication standards: realist syntheses. BMC Med. 2013;11:21.

**SECTION**			**Page # in Manuscript**
**TITLE**			
1	In the title, identify the document as a realist synthesis or review		*Title page*
**ABSTRACT**			
2	While acknowledging publication requirements and house style, abstracts should ideally contain brief details of: the study's background, review question or objectives; search strategy; methods of selection, appraisal, analysis and synthesis of sources; main results; and implications for practice.		*page 2*
**INTRODUCTION**			
3	Rationale for review	Explain why the review is needed and what it is likely to contribute to existing understanding of the topic area.	page 3 - Introduction
4	Objectives and focus of review	State the objective(s) of the review and/or the review question(s). Define and provide a rationale for the focus of the review.	page 3 - Introduction
**METHODS**			
5	Changes in the review process	Any changes made to the review process that was initially planned should be briefly described and justified.	No. Process as outlined in the protocol.
6	Rationale for using realist synthesis	Explain why realist synthesis was considered the most appropriate method to use.	page 3 - Introduction
7	Scoping the literature	Describe and justify the initial process of exploratory scoping of the literature.	page 4, 5 – Methods + described in protocol
8	Searching processes	While considering specific requirements of the journal or other publication outlet, state and provide a rationale for how the iterative searching was done. Provide details on all the sources accessed for information in the review. Where searching in electronic databases has taken place, the details should include, for example, name of database, search terms, dates of coverage and date last searched. If individuals familiar with the relevant literature and/or topic	page 5 – Systematic search

SECTION			Page # in Manuscript
**TITLE**			
		area were contacted, indicate how they were identified and selected.	
9	Selection and appraisal of documents	Explain how judgements were made about including and excluding data from documents, and justify these.	page 5 – Stage 3 Data appraisal
10	Data extraction	Describe and explain which data or information were extracted from the included documents and justify this selection.	page 5 – data extraction
11	Analysis and synthesis processes	Describe the analysis and synthesis processes in detail. This section should include information on the constructs analyzed and describe the analytic process.	page 5-6 – Development of refined programme theories
RESULTS			
12	Document flow diagram	Provide details on the number of documents assessed for eligibility and included in the review with reasons for exclusion at each stage as well as an indication of their source of origin (for example, from searching databases, reference lists and so on). You may consider using the example templates (which are likely to need modification to suit the data) that are provided.	Figure 2
13	Document characteristics	Provide information on the characteristics of the documents included in the review.	page 7 – results
14	Main findings	Present the key findings with a specific focus on theory building and testing.	pages 7-18 – results
DISCUSSION			
15	Summary of findings	Summarize the main findings, taking into account the review's objective(s), research question(s), focus and intended audience(s).	pages 19-21 – discussion

SECTION			Page # in Manuscript
**TITLE**			
16	Strengths, limitations and future research directions	Discuss both the strengths of the review and its limitations. These should include (but need not be restricted to) (a) consideration of all the steps in the review process and (b) comment on the overall strength of evidence supporting the explanatory insights which emerged.<br><br>The limitations identified may point to areas where further work is needed.	page 21 - strengths & limitations
17	Comparison with existing literature	Where applicable, compare and contrast the review's findings with the existing literature (for example, other reviews) on the same topic.	pages 19-21 – discussion
18	Conclusion and recommendations	List the main implications of the findings and place these in the context of other relevant literature. If appropriate, offer recommendations for policy and practice.	page 21 – conclusion
19	Funding	Provide details of funding source (if any) for the review, the role played by the funder (if any) and any conflicts of interests of the reviewers.	Title page

Document 11: CMOCs table

Study ID	Intervention name	Intervention design features	Context(s)	Mechanism(s) - Resource	Mechanism(s) - Reasoning	Outcome(s)	If-then-because statement (CSMOC)	Relevant IPT (protocol)
Ainul2022	Keeping Girls in Schools (KGIS)	School capacity building - teachers and classroom equipment; mobile classroom (COVID)	Schools with limited resources	Capacity building of teachers, laptops, internet, projector in school	Capacity building of school and teachers will sustain the intervention even after it ends. This may provide resources to all adolescent girls and lead to overall gains in education and health outcomes. The school context and prevalent social norms have a positive change.	In intervention areas - Intermediate : decrease in child marriage, decrease in school dropout due to CM, increase in contraceptive use	1. If schools in low-resource settings (C) are provided capacity building in the form of teacher training to better equip them to provide needs-based support in subjects like Maths and English, classroom equipment like laptops, internet and projector and mentor support to discuss gender-related inequality (M-Res) then the school will see overall gains in education and health outcomes (IO) because teachers will be more involved with students to discuss their problems in various subjects and mentors will be able to discuss topics of SRH which will reduce stigma on these topics. The students will be interested in learning and will regularly come to school to use the new equipment.	Material resource in school - social norms (new)

							This will create positive social norms changes in the school environment (M-Rea) which acts as a safe space for girls.	

		Classes with trained teachers and sessions with mentors	Girls underperforming secondary school	Digital learning (virtual and multimedia), need specific education material	Digital learning resources and need-specific resources for girls ensure knowledge uptake and subsequent increased interest in learning due to the newness of the methods, problem based learning, and ability to follow content - leading to retention of knowledge and application in daily life		2. If underperforming adolescent girls in school (C) are provided with need-specific remedial education using digital media and problem-based learning resources in both in-person and online formats (M-Res) - then school dropout will reduce (IO) - because new learning methods may lead to higher attention span, interest in class material and retention of knowledge (M-Rea) which will improve the girl's performance at school. Families of girls performing well at school may, as a result, decide to keep them in school longer and may postpone child marriage, and this subsequently reduces adolescent pregnancy (O).	Digital Education - keep girls in school (new)

		Weekly meetings with mentors, peers	Out of school girls, girls in school	Mentorship and role model in community - young women - provided constant support, weekly group meeting	Mentors will act as role model - provide motivation and increase aspiration to do new things	Main: decrease in adolescent pregnancy	3. If vulnerable adolescent girls both in school and out of school (C) are provided life skill sessions, including computer training and basic health services skills with female mentors from the community in a safe group setting (M-Res), then they may be involved in new job opportunities, may study more, postpone marriage and pregnancy (O) because **girls** will experience low social isolation/ build social networks, gain problem-solving skills, gain technical know-how, get regular support from friends and mentors which will lead to an increase **in** human and social capital and **they** will have improved decision making capacity (M-Rea).	IPT 5, 2, 4, 7 (life skill education + safe space + mentor)

		Meetings with mentors, peers	Out of school girls with financial responsibilities in the family	Skill building session on computer skills, finance literacy, health services	Skill session on computer, health and finance literacy will increase the knowledge of girls on these topics, will give them alternative ideas about income generation with updated technical skills required in today's world - learning by doing will help retain and apply knowledge - increase human capital and social network Regular meetings with peers and mentors will reduce social isolation - will give girls a place to share their ideas, thoughts and feelings, this will increase social connectedness and build trust - overall increase in social capital		4. If out-of-school girls (C) are provided financial education by a female mentor from the community combined with cash transfers for the most vulnerable girls (M-Res), then they may have more livelihood opportunities, which may lead to postponement of marriage and subsequent pregnancy (O) because they will have the job-relevant skills - learning by doing will help them retain and apply knowledge - which will increase their human capital. They will have decreased economic dependence on their families which may increase their decision making capacity. Regular meetings with mentor will also increase social connectedness and give girls the opportunity to form a social network reducing social isolation	IPT 1, 3

				Direct cash transfer to most disadvantaged girls with economic responsibilities	Cash transfer will provide resources to families to keep girls in school - economic empowerment of girls and family - opportunity cost - dependence on parents slightly reduced		and find gainful empolyment (M-Rea).	
		Family and community meeting	High child marriage	Family and community engagement - awarness on CM, health, education, employment	Meeting with family and community will increase awareness on adverse effects of CM - will increase understanding of girls lived realities - this will increase support for education, skill building and shift social and gender norms to support girls		5. If families in communities with high child marriage prevalence (C) are engaged in group meetings with teachers and mentors on adverse effects of Child Marriage on adolescent girls, like impact on health, education, and employment opportunities (M-Res) then child marriages may reduce (O) because the people in the community will have an increased understanding of the lived realities of girls - which will increase support for education, skill building and shift social and gender norms to support girls and their future aspirations (M-Rea).	Community engagement (new), IPT 6

Ajayi2021	In their Hands (t-safe) program	Trained community health volunteers conducting mobilization, peer network	Counties with highest rates of teenage pregnancy	Community sensitization on adolescent SRH and a radio program focused on parents to promote communication between parents and their adolescents.	Support: Community based awareness programs and parental discussions on SRH needs - create a supportive environment where adolescents can talk to their parents without judgment - this creates an environment where girls feel supported to make the right choice. Gap filled the Tenuous nature of their relationship situation.	Intermediate : Sexual activity and relationships ; SRH knowledge; contraceptive knowledge, access, choice and contraceptive decision-making - use. Exposure to family planning messages	6. If families living in high teenage pregnancy, STI and HIV prevalent areas (C) are targeted for community sensitisation on adolescent SRH through a radio program on non-judgemental communication between parents and adolescents on sexual health (M-Res) then parents and adolescents will discuss SRH topics freely, adolescents' SRH knowledge will increase, and their contraceptive knowledge will increase subsequently leading to increase in contraceptive use and reduction in adolescent pregnancy (O) because adolescents will have a supportive environment in their families and communities where they can discuss sensitive topics, and have the power to make the right choice for themselves.	Community engagement/gender social norm change (new), IPT 6

**Wado2020**		Phone and app to access information and services	Highest unmet need for contraception among adolescents	Digital platform that gives access to information on SRH, free access to to youth-friendly facility - enrolling either self or via ref of a friend	Need: when girls have access to a digital platform (in their control, anonymous) with information on SRH and how to access free services they value their sexual health and want explore these services as her own choice. Gap filled: Misinformation and misconceptions regarding contraceptives' side effect like infertility. Lack of information on how to prevent pregnancies before sexual debut	Main: adol preg	7. If adolescent girls living in high teenage pregnancy, STI and HIV prevalent areas (C) are provided access to a digital health platform/app with information on SRH, free access to youth health facilities, and pharmacies selling health products (M-Res) then adolescents will increase their use of contraceptives, decrease unprotected sex, test for pregnancy and STI (O) because they have control over what type of information they access decreasing misinformation and misconception, how they access it and keep it anonymous thourgh the app. They also have the choice to visit the health facility or pharmacy to get health products without being dependent on elders for funds. This will increase their overall sexual health agency and choice (M-Rea).	Digital health (new)

		Youth friendly facilities and pharmacies	High rates of new STIs and HIV	Services like counseling for contraception and HIV self-testing, contraceptives, and pregnancy testing.	On my terms: When SRH services on contraceptive methods and HIV or preg testing are provided by youth friendly orgnanization (private or NGO) that understand girls' needs and do not stigmatize them then girls are enabled to choose their contraceptive methods or tests on their terms and holding providers accountable. Filling gap: Lack of trusted people to counsel young girls confidentially on sexuality issues in general and pregnancy prevention.		8. If adolescents (both boys and girls) living in high teenage pregnancy, STI and HIV prevalent areas (C) are provided free health products like counselling for contraceptives, HIV self-test kits, contraceptives and pregnancy testing at a youth-friendly health facility (M-Res) then they will increase the use of contraceptives and tests, decrease unprotected sex which will subsequently reduce adolescent pregnancy (O) because they will have access to information from a trusted health professional who understands their needs, does not stigmatize the subject, and keeps the information confidential. The free access also reduces financial dependence on parents and gives adolescents the power to make their own choices.	IPT 6

**Baird2019**	**Education, cash transfer, and Health Risk (SIHR)**	Cash transfer at a local facility	Agricultural area, poor background, informal jobs, few formal job opportunities. In school and out of school girls at baseline.	Cash grants to in school and out of school girls	the causal pathway to improved welfare over the long run is more likely to be human capital accumulation, either in the form of education and skills or health especially reproductive and sexual health for adolescent females.	marriage, school dropout, SRH knowledge, labor market skills	9. If adolescent girls belonging to poverty-stricken areas with low school retention, low formal employment opportunities and high incidence of child marriage and teen pregnancy (C) are provided unconditional cash transfers, then their families might have increased financial capacity to either send them to school or to invest in their employability or use it for other purposes - which may (in short term) or may not (long term) lead to decrease in child marriage and adolescent pregnancy because there is no clear pathway to ensure that the benefit goes to the girls.	IPT 1, 3 (economic incentive and financial training)

			Low secondary school completio n. High child marriage, teen pregnancy		UCTs increase school enrollment indicates that poverty is an important cause of school dropout in this population, and that poor parents will invest at least some of the additional funds from a positive income shock towards the education of their daughters. Women's agency, intra-household bargaining power, and empowerment.	Main: adolescent pregnancy	10. If school-going girls belonging to poverty-stricken areas with low school retention, low formal employment opportunities and high incidence of child marriage and teen pregnancy (C) are provided conditional cash transfers based on their school attendance early on (M-Res), then their school dropout decreases, SRH knowledge increases. Subsequently, incidence of child marriage and adolescent pregnancy also decreases (O) because parents may invest at least some of the additional funds towards the education of their daughters; they will keep the girls in school for longer beacuse of additional financial resources that can be used for daily needs thus reducing the financial burden on parents; school retention will also lead to the accumulation of knowledge and skills for	IPT 2

							employment, girls may utilize these skills and employment as negotiation to delay marriage (M-Rea).	

Burke2022	ES and HIV	Course on financial planning in school or community facility	Orphans and vulnerable children (OVC), people living with and affected by HIV/AIDS, Pregnant and/or HIV-positive adolescents (both girls and boys)	Course on budgeting and saving, education on different savings options, and earning money	Combined programmes build skills to improve financial well-being, women's empowerment and gender equity, and thus reduce vulnerability to HIV. Our theory of change proposed that an HIV-prevention intervention that increases mental resources by teaching adolescents about safe sex practices and builds communication and negotiation skills would lead to safer sex practices. Economic interventions can be used to reduce economic vulnerability, by increasing tangible resources - a key structural factor	Intermediate: sexually transmitted infections, self-reported economic and sexual behaviours/knowledge	11. If vulnerable adolescents at high risk of HIV, living in disadvantaged communities (C) are provided with economic strengthening courses on budgeting, saving and earning money (M-Res) then their knowledge of economic opportunities and financial knowledge may not increase (O) because they do not have an additional intervention component to apply this knowledge that will aid retention of knowledge (M-Rea). Also, as they live in disadvantaged communities without channels linking them to economic opportunities (M-Rea) - they will not find gainful employment. So, the knowledge gained will not convert into human capital (M-Rea) and, therefore, not lead to changes in health outcomes.	no effect CSMOC, IPT 1, 3

		Course on broad range of risky sexual behavior in school or community facility		Course covered topics including dealing with loss and grief, decision-making, drugs and alcohol, HIV and other STIs, healthy relationships, communication skills, safer sex and contraception	contributing to risky sexual behaviours	Main: adolescent pregnancy	12. (If) vulnerable adolescents at high risk of HIV, living in disadvantaged communities (C) are provided with HIV risk reduction specific health education, including information on HIV/AIDS, healthy communication, safe sex, decision making and substance abuse (M-Res), then their risk of adolescent pregnancy (O) may not reduce because the intervention only focuses on the individual and not their environment. The adolescents in disadvantaged communities may not have the power to use the skills learned in the course if negative community norms prevail (M-Rea). They might not have access to resources to buy health products because of a lack of funds and opportunities to gain employment (M-Rea) - thereby limiting the transformation of skills into positive outcomes.	no effect CSMOC, IPT 8

Dupas2012	Sugar daddy	In school session with trained male teachers and trained female external consultants	High rate of HIV prevalence among 15-24 women	Training of teachers on different types of HIV prevention and Interaction between students and teachers in a classroom setting.	Teachers were better equipped to answer questions that might arise on HIV transmission. They felt more comfortable talking about the topic. They had higher awareness about the topic.<br>Interaction between students and teachers increased the understanding of HIV transmission. Students feel comfortable with the existing teachers and trust them to provide correct information.	Intermediate: knowledge about HIV, HIV prevention plans, self-reported sexual behaviour	13. If school-going girls living in areas with high school dropout, HIV, early sexual debut, and teen pregnancy prevalence (C) are provided HIV prevention abstinence education in the classroom by trained teachers (male) (M-Res) then there may be a reduction in unprotected sex and subsequently in adolescent pregnancy (O) because teachers are well equipped to answer questions and feel comfortable doing so with girls in a classroom setting (M-Rea). This will increase interaction between teachers and students, leading to higher knowledge on the topic and improving trust and communication between them (M-Rea).	IPT 8, 2 (HIV education)

		Most teachers (70%) were male	Girls become sexually active at a younger age, Teen pregnancy was much more frequent	Interaction between female external consultant and students using videos. Interaction between students in the presence of trainer in a classroom setting. Presentation and video on relative risk awareness of having sex with older men.	Students felt comfortable with extrnal consultant beacuse of teachers' status and gender in relation to pupils, and discomfort in discussing sensitive topics. Videos and presentation (digital tools) help students visualize the information provided and increase information retention.	Main: adolescent pregnancy	14. If school-going girls living in areas with high school dropout, HIV, early sexual debut, and teen pregnancy (C) prevalence are provided HIV prevention sexual health education using digital media and relative risk messaging in the classroom by trained female external consultants (M-Res) then girls will have improved risky sexual behaviour, increase in contraceptive use and subsequent reduction in adolescent pregnancy (O) because the female consultant can connect with girls better (M-Rea) in comparison to male teachers both in terms of gender and power differential in classroom. Girls may feel more comfortable discussing sensitive topics (M-Rea) with someone new as it maintains privacy (M-Rea) in their immediate environment. The use of digital media may also help students visualise the	IPT 8, 2, Digital health

							information provided and increase information retention (M-Rea). Relative risk messaging promotes critical thinking about sexual decision-making and gives girls the power to choose (M-Rea) instead of abstinence-focused messaging, where they have only one choice (M-Rea).	

		Only girls	High school dropout	Hour long in class quiz on HIV knowledge, sexual behaviour of peers, beliefs on risk of adol preg and HIV infection, own behaviour.	Interaction between peers also increases social comparison and conformity - if most peers think that getting pregnant before 18 is not suitable, that will influence others. On the other hand, if most peers think otherwise, that might lead to peer pressure to indulge in risky practices. But the presence of a trainer can control the spread of misinformation.		15. If school-going girls living in areas with high school dropout, HIV, early sexual debut, and teen pregnancy (C) prevalence are provided sexual health education in a group setting in the classroom by trained teachers or consultants (M-Res), then girls will have improved risky sexual behaviour, increase in contraceptive use and subsequent reduction in adolescent pregnancy (O) because interaction between peers will increase social comparison and conformity (M-Rea). If most peers think that getting pregnant before 18 is not suitable, that will influence others. On the other hand, if most peers think otherwise, that might lead to peer pressure (M-Rea) to indulge in risky practices. However, the presence of a trainer may control the spread of misinformation (M-Rea).	IPT 8, 2

					Quiz help evaluate own thoughts and beliefs on the topic. It helps plan for suitable outcome and may lead to setting ideas on what not to do. Being unanonymous - there is no risk of stigma and students can be true to what they think.		16. If school-going girls living in areas with high school dropout, HIV, early sexual debut, and teen pregnancy (C) prevalence are asked to take a test/quiz on personal and peer sexual health behaviours in a group setting in the classroom by trained teachers or consultants (M-Res) then girls will have improved risky sexual behaviour, increase in contraceptive use and subsequent reduction in adolescent pregnancy (O) because the quiz will help them evaluate their own thoughts and beliefs (M-Rea) on the topic. It will help them with goal setting and planning for suitable outcomes (M-Rea) and may lead to setting behaviours (M-Rea) on what not to do. Quiz being anonymous - there is no risk of stigma and students can be true to what they think (M-Rea).	IPT 8, 2

Hensen2022 Hensen2021	Yathu Yathu	Community-based, peer-led spaces, called Yathu Yathu hubs, that provide comprehensive SRH services	Boys and girls	Community-based, peer-led spaces, that provide comprehensive SRH services including HIV testing, counselling, referrals, contraceptives, comprehensive sexuality education and edutainment sessions (MTV Shuga and Love Games), linked to health facility via referals	Community spaces that are easy to access and youth-friendly may increase adolescents' trust in the health system. They may also provide a safe space to talk about sexual health with peers, counsellors and friends - which may motivate them to visit the hub more regularly. The socialisation aspect may reduce stigma around the topic and increase uptake of services.	Intermediate: knowledge of HIV status, uptake of HIV testing, uptake of HIV treatment and prevention services, alcohol use, history of contraceptive use, condom use, and expectations for the future	17. If adolescents (boys and girls) in periurban communities with high prevalence of HIV (C) are provided access to trained health professional run, youth-friendly hubs with SRH services and products, digital CSE and referral to health facilities (M-Res) but amidst a public health emergency (COVID) (C) then their SRH knowledge, uptake of services, contraceptives and subsequent adolescent pregnancy may not reduce (O) because even though they have a safe space to discuss sensitive topics with professionals and peers, have positive reinforcement from the	No effect CSMOC, IPT 6

		Loyalty card to accrue points for accessing SRH services and spend points on rewards	Periurban communities with access to health facilities	“loyalty cards” that allow AYP to accrue points for accessing SRH services and spend points on rewards including toothpaste, toothbrush, and soap, but also nail polish, vouchers for barber/hairdresser, and reusable menstrual pads.	Loyalty cards may activate positive reinforcement by getting a reward for a behaviour change (use of SRH services). This may make the participants more likely to repeat the behaviour to receive the favourable rewards again. The rewards' economic aspect might also appeal to participants with limited resources. This reduces their dependence on parents and partners to buy the products they need. This also gives them a sense of self-sufficiency to take control of their health decisions.	Main: adolescent pregnancy	loyalty card to change behaviour and have independence from guardians in buying health products these spaces might be closed for extended periods and might be out of stock of various health products which may have led to loss of confidence and trust in the services provided (M-Rea).	

		Community engagement activities to provide info on avaiable health services - community mobilizers	Prevalence of HIV	Community engagement activities to inform AYP and the broader community of the services available through Yathu Yathu.	The community engagement activity might target changing the social norms and stigma around SRH. Discussing these topics and providing products related to this might induce proactive thinking about it. This may reduce the social barriers to accessing services.		18. If periurban communities with a high prevalence of HIV (C) are provided community engagement activities by trained community mobilizers on SRH topics and services available for adolescents (M-Res) then social norms and stigma around these topics will reduce leading to increased communication and support between parents and adolescents - further leading to positive sexual behaviour change (O). But in case of a public health emergency, these activities will be stopped, and **and** norm shift will not take place (M-Rea). Thus leading to no impact on health.	Community engagement (new)<br>No effect CSMOC, IPT 6

		Youth friendly health services - peer support workers (PSWs), lay counsellors and nurses			Hubs closed for 3 months in response to COVID-19 in mid-2020 and stock-outs of oral contraceptives at the hubs and health facilities between 2020 and 2021, it is possible that some AGYW discontinued use of hormonal contraceptives, particularly the pill, and subsequently lost confidence that services would be offered consistently. Mobilisation activities were insufficient to shift these norms.			

Austrian2019	AGEP	Weekly group meeting with trained young female mentor	Urban and rural site - close proximity to health centers and bank	Information on SRH, HIV, nutrition; interaction with mentors, access to health services with health voucher	Health assets: knowledge (SRH, HIV, nutrition), aspirational (staying STI free), efficacy (confidence with providers), access (health voucher)	Intermediate: educational attainment, sexual debut, STIs, ability to support themselves and their families financially, and control over health and financial decision-making.	19. If the most vulnerable girls from lower-income backgrounds in both urban and rural areas (C) are provided comprehensive sexual and reproductive health education in a safe space in the community and free access to health services at youth-friendly facilities (M-Res), even then they might not have positive changes in sexual behaviour, GBV and adolescent pregnancy (O) because the most vulnerable girls might not be able to attend the sessions on SRH at the club due to travel restrictions from family, resource constraints to reach the place, responsibilities at home, job, sessions being not culturally appropriate, or they do not like the group setting due to privacy issues (M-Rea) thus leading them to not access the health service either.	No effect CSMOC IPT 6, 5, 2, 4, 7

**Hewett2014**		Safe spaces	High prevalence of adolescent pregnancy, child marriage, school dropout, sexual and physical violence was high, SRH knowledge low	Skills in communication, leadership; discussion on gender roles, rights, and relationships; weekly meetings with peers	Social assets: knowledge (communication, leadership), aspirational (gender roles, rights), efficacy (relationships, safety net), access (weekly meetings)	Main: adolescent pregnancy	20. If the most vulnerable girls from lower-income backgrounds in both urban and rural areas (C) are provided life skill training by trained female mentors from the community in a safe space at the community even then they might not have positive changes in sexual behaviour, GBV and adolescent pregnancy (O) because the most vulnerable girls might not be able to attend the sessions at the club due to travel restrictions from family, resource constraints to reach the place, responsibilities at home, job, sessions are not appropriate for their needs, or they do not like the group setting due to privacy issues (M-Rea) thus limiting the translation of session into knowledge, aspiration and self efficacy.	No effect CSMOC IPT 6, 5, 2, 4, 7

**Hewett2017**		Health voucher	lower-income backgrounds and live with multiple levels of vulnerability, e.g., physical and social isolation, living without parents, living in low-income households, and not attending school.	Course on earning and saving money, goal setting and reaching goals, confidence in money management, access to bank account and knowledge on how to use the bank services.	Economic assets: knowledge (earning and saving money), aspirational (reaching goals), efficacy (confidence in money management), access (bank account)		21. If the most vulnerable girls from lower-income backgrounds in both urban and rural areas (C) are provided financial education by trained young mentors from the community in a group setting and access to a savings bank account, even then they might not have positive changes in knowledge on financial decision making, sexual behaviour, GBV and adolescent pregnancy (O) because they might not participate in the program or attend the session due to pre-existing barriers (see above) (M-Rea) even though these sessions and	IPT 3, 1<br>No effect CSMOC

**Austrian2018**		Savings account		Saving account	While the girl's groups provided capacity building in money management, budgeting and savings, the provision of a bank account was designed to provide a mechanism for knowledge and skills to be operationalized in practice. it was hypothesized to reinforce girls' money management skills, promote economic asset building, grow a culture of savings, facilitate economic independence and provide assets in cases of emergencies or other basic needs.		account are targeted to increase their economic asset.	

					No effect on pregnancy: a) a large proportion of the (vulnerable) girls invited to the programme did not participate - maybe because they had other barriers to overcome<br>b) among those who did participate, only a sub-segment of them participated actively in the safe-spaces sessions. - maybe they still do not feel comfortable sharing - linked to group environment? different opinion?			

**Makino2021**		Safe spaces	High prevalence of adolescent pregnancy, child marriage, school dropout, sexual and physical violence was high, SRH knowledge low	Trained young mentors from the community. Interaction between mentor and girls in safe space - group setting. Age and marital status segregated groups. Safe space to discuss essential experiences of the past week and Information on health, life skills, and financial education topics	Interacting with trained young mentors (who will act as role models for girls) in the community will be an example for girls of what opportunities they might get apart from the gender-specific roles ascribed to them by the society. This can also act as a motivation to set and achieve ambitious goals. Age and marital status-segregated safe spaces will reduce the stigma associated with sensitive topics as girls will be with peers in a similar setting and phase of life. They will bring a sense of emotional support and shared experiences and reduce social isolation.	Intermediate : sexual violence and premarital sex	22. If uneducated girls with similar demographic characteristics like age and marital status in communities with high sexual violence and high premarital sex prevalence (C) are provided trained mentor-led life skill training in a safe space with peers (M-Res) then early pregnancy is delayed or prevented (O) because discussing SRH topics with peers and mentor may increase knowledge and reduce the stigma associated with these topics and bring a sense of emotional support and shared experiences that further reduces isolation (M-Res). This may motivate them to set positive health and life goals for themselves and increase their decision-making power over their own sexual behaviour (M-Rea).	IPT 5, 2, 4, 7 (life skill education + safe space + mentor)

		Health voucher	lower-income backgrounds and live with multiple levels of vulnerability, e.g., physical and social isolation, living without parents, living in low-income households, and not attending school.	Health voucher covering essential health exams, age appro srh services	Information on health life-skills and financial education topics will build girl's knowledge - build assets or human capital for them to make informed decisions and set realistic goals	Main: Adolescent pregnancy	23. If uneducated girls in communities with high sexual violence and high premarital sex prevalence (C) are provided health vouchers giving access to free health services at a youth-friendly facility (M-Res) then early pregnancy is delayed or prevented (O) because this may encourage them to access health services without financial dependence on parents, or partners, which in turn increases their decision-making power over their own sexual behaviour (M-Rea).	IPT 6, health products
		Savings account	Urban and rural site - close proximity to health centers and bank	Savings account	Health vouchers will give girls access to healthcare that they can access without financial dependence on parents or partners - this will give them the power to make decisions and take control of health needs			

		Weekly group meeting with trained young female mentor			Savings account may give girls a place to save money and set financial goals. She can apply the learnings from the training and retain that knowledge by practising the techniques taught - this might lead to an increase in confidence.			
Mbizvo2023	**CSE-Health Facility linkages**	CSE	In school sessions after consulatio n with parents	In class CSE education and adolescent SRH services in facility catchment areas	CSE increases knowledge about healthy behaviours	Intermediate : age at sexual debut, unprotected sex, health seeking behaviour for SRH services, demand for SRH services, SGBV		

		Youth friendly facility	Districts with high adolescent pregnancy rates.	Health outreach in school via health fairs, training of teachers and health workers in youth-friendly approaches, anonymous referral, health services provision like HIV counseling and testing, information on pregnancy prevention and pregnancy testing, contraceptive counseling, HIV prevention education, menstrual health and personal hygiene promotion, and hypertension and obesity monitoring.	Health fairs give access to information on school grounds in the presence of other peers who might also visit the stalls with information together - creating a peer environment, and anonymous referral create autonomy and security for the participants to access services without the threat of exposing their identity - this increases confidence in getting the care required	Main: adolescent pregnancy	24. If adolescents (boys and girls) in school living in high pregnancy prevalence areas (C) are provided comprehensive sexual health education in class along with health fairs on school grounds and anonymous referral slips to a youth-friendly facility by trained health providers where they can get free testing, counselling and health monitoring (M-Res) then there is a reduction in unwanted adolescent pregnancy (O) because CSE and health fairs give access to information on school grounds in the presence of other peers who might also visit the sessions and stalls together - creating a supportive peer environment, and anonymous referral creates autonomy and security for the participants who want to access services without the threat of exposing their identity - this increases confidence in getting the care required translating	IPT 6, health products

							into uptake of services (M-Rea).	

		Linkages between school and health facility		Health outreach outside of school via referral services, training of teachers and health workers in youth-friendly approaches, provision of services like HIV/STI prevention, puberty, personal hygiene, menstrual health, and adolescent pregnancy prevention	Health outreach outside of school at youth friendly facility with trained health providers may reduce stigma around the topic of risky behaviour - increasing trust. Provision of free health services may increase demand and aid risk reduction. Independent visit to the facility may create autonomy and security for the participants to access services without the threat of exposing their identity.		25. If adolescents (boys and girls) in school living in high pregnancy prevalence areas (C) are provided comprehensive sexual health education in class along with access to health services outside of class at a youth-friendly facility where they can get free testing, counselling and health monitoring (M-Res), then there is a reduction in unwanted adolescent pregnancy (O) because CSE and access to free care may reduce stigma around the topic of risky behaviour - thus increasing trust in the system - that increasing uptake of available services. The provision of free health services may increase demand and aid risk reduction. Independent visits to the facility may create autonomy and security for the participants to access services without the threat of exposing their identity. (M-Rea).	IPT 6, health products

		Health services		Community engagement on health services available	Community engagement reduces negative gender and social norms which in turn, creates a judgement-free atmosphere to access care - increase in confidence, trust and sense of belonging - uptake of services - risk reduction - reduction in pregnancy		26. If communities in high pregnancy districts (C) are provided with information and community engagement activities on health services available for adolescents at youth-friendly facilities (M-Res), then stigma and negative social norms around SRH will be reduced subsequently leading to uptake of services - risk reduction - and reduction adolescent pregnancy (O) because this will create a judgement-free atmosphere to access care for adolescents - increase in confidence, trust and sense of belonging for them in the community, and improve communication between parents and children (M-Rea).	IPT 6, health products

Oberth2021	Sista2Sista	Life skills with interactive activities with trained mentors	Girls are at high risk in five key areas: self-awareness, education, social relationships, sexual knowledge and financial awareness.	Girls-only club, mentoring, a course on communication, gender and power, family planning, sexually transmitted infections, HIV, stigma and discrimination, menstrual health, cancer awareness, consent, SGBV, finance management, health topics, individual sessions, trauma counselling, the interaction between mentor and girls, and between girls.	Clubs create a safe space for girls to talk about sensitive topics - increase trust, belonging, reduction in isolation; interaction between girls and mentors also create healthy relationships for girls, mentor may act as a role model - increasing in aspirations, motivation, and emotinal support; courses in club increase knowledge and skills - increase confidence and self-efficacy - increase in decision making power and judgment for healthy choices	HIV testing, marriage, school attendance, reporting sexual abuse, family planning	27. If vulnerable girls with low education, self-awareness, sexual knowledge and financial resources (C) are provided life skills training on a variety of health, finance and empowerment topics at a safe space in the community by a trained mentor (M-Res) then for girls who attend all the sessions adolecent pregnancy reduces (O) because clubs create a safe space for girls to talk about sensitive topics - which increases trust, belonging, reduction in isolation; interaction between girls and mentors also create healthy relationships for girls, mentor may act as a role model - increasing in aspirations, motivation, and emotional support; courses in club increase knowledge and skills - increase confidence and self-efficacy - increase in decision making power and judgment for healthy choices (M-Rea).	IPT 5, 2, 4, 7 (life skill education + safe space + mentor)
		Safe spaces				Adolescent pregnancy		

Wang2005	CSE	Comprehensive sexual health education with digital components: distribution of educational reading materials, screening of educational videos, lectures, peer group discussions	In school and out of school Boys and girls	The intervention used six types of activities to provide information and services regarding abstinence, sexuality, contraception and HIV/AIDS prevention: distribution of educational reading materials, screening of educational videos, lectures, peer group discussions, and provision of reproductive health services and counselling. Nine brochures, pamphlets, and four books were distributed to each participant in the intervention group during the 20-month study period.	CSE increases knowledge about sexual health-related topics, with new knowledge comes a sense of judgement to make healthy choices, Digital media use, lectures and group discussions helps with knowledge retention, inculcates a group learning environemnt and sense of belonging with peers	first and most recent intercourse, contraceptive use, experience of sexual coercion, and induced abortion	28. If adolescent and young boys and girls both in and out of school living in suburban areas with high cohabitation, early sexual debut and adolescent pregnancy (C) are provided with comprehensive sexual health education with digital media, educational resources, lectures and peer discussions (M-Res) then their coercing a partner into having sex decreases and use of contraceptives increases but no change in adolescent pregnancy (O) because CSE increases knowledge about sexual health-related topics, and with new knowledge comes a sense of judgement to make healthy choices. Digital media use, lectures and group discussions help with knowledge retention and inculcate a group learning environment and a sense of belonging with peers (M-Resa).	Digital Education

		Provision of reproductive health services and counselling, Access to contraceptives	high proportion of resident adolescents be sexually active, higher rates of premarital pregnancy and induced abortion, high cohabitation	Peer group discussions	Add	pregnancy	29. If adolescent and young boys and girls both in and out of school living in suburban areas with high cohabitation, early sexual debut and adolescent pregnancy (C) are provided reproductive health services and products (M-Res) then use of contraceptives increases but no effect on adolescent pregnancy (O) because access to health services and counselling reinforces learning by application of skills learnt in the course, availability of free contraceptives and tests empowers participants to make decisions and use services accordingly (M-Rea).	No effect CSMOC, IPT 6
		Credentialed female counselor	Suburban area with well-established family planning commission	Provision of reproductive health services and counselling.	Access to health services and counselling reinforces learning by application of skills learnt in the course, availability of free contraceptives and tests empowers participants to make			

					decisions and use services accordingly.			

Aventin2021	If I were Thabo	With boys - in and out of school	In school - for students	Slides, classroom activities, and homework exercises that involve adolescents' parents. Homework activity an individual activity that helps young people to safely identify an older trusted adult that they could speak to about SRH. Parent/caregiver activities are address local concerns, particularly addressing cultural taboo relating to adults speaking to children about SRH and increasing knowledge that talking about SRH does not encourage sex. Parent/caregiver/educator materials refer to SRH education rather	Beliefs about consequences: positive planning, anticipated regret, risk perception Attitudes and beliefs: contraceptive use, barriers to use, harmful norms Knowledge: avoiding HIV, condom use myths, local SRH support, sexual readiness, transactionl sex Skills: obtain and use condoms, communication and negotiation with partners, peers, parents, professionals ---> Social influences challenging gender norms, peer norms, social norms Beliefs about capabilities - perceived behavior control and self efficacy ---> intentions to avoid unprotected	delayed initiation of sexual intercourse and/or consistent use of contraception, STI/STD transmission	30. If young boys both in school and out of school living in high HIV and adolescent pregnancy areas (C) are involved in a group-based, context-specific interactive drama showcasing a young couple's journey with adolescent pregnancy (M-Res) then it may change their ideas about gender roles and responsibilities, may lead to change in beliefs about using contraceptives, may lead to delayed initiation of sexual intercourse and subsequently incidence of adolescent pregnancy (O) because they may gain knowledge and skills on how to use contraceptives, where to acquire it and how to avoid pregnancy; they will be able to perceive risky situations and plan accordingly to avoid pregnancy; and they will also know how to discuss these topics with their partners and peer to	Digital health education (new)

				than relationships and sexuality education.	sex, plan for positive sexual relationship when ready		better negotiate for themselves (M-Rea).	

**Skeen2022**		Interactive film and radio drama version	In communit y - for drop outs	Interactive context-specific film in group setting with discussion component on 'Controversial statements' looking at gender stereotypes, 'People and things' looking at roles of men and women and how we treat them.	It is designed to promote critical thinking and questioning about the social pressures that normally situate teenage pregnancy and its prevention as a female-only issue.	adolescent pregnancy	31. If young boys both in school and out of school living in high HIV and adolescent pregnancy areas (C) are involved in a group-based, context-specific activities that involves identifying gender bias, roles and responsibilities and home based activities to identify trusted adults to discuss SRH topics (M-Res) then it may change their ideas about gender roles and responsibilities, may lead to change in beliefs about using contraceptives, may lead to delayed initiation of sexual intercourse and subsequently incidence of adolescent pregnancy (O) because they may gain knowledge on how to maintain healthy and trusting relationships, they may gain skills to negotiate and communicate with partners about SRH topics, they may find advice from trusted adults, and they may question social pressure around	Peer and social norm change

							adolescent pregnancy which may give them the confidence to apply this in their lives to prevent unwanted pregnancy (M-Rea).	

			Vulnerable adolescents - high HIV and adol pregnancy; Gender inequitable setting		Activities catering to: Knowledge: know how to evaluate relationships; know where to seek help for unhealthy or abusive relationships. Know what is consensual and non-consensual sex; know about the possible negative consequences of transactional sex. Skills: communicate expectations with sexual partners; avoiding or leaving unhealthy relationships Beliefs about consequences: believing that unhealthy relationships will impact negatively on current life and future goals Believing that gender-equal relationship can be happy and fulfilling. Self-efficacy: Confidence in ability		32. If parents, caregivers and teachers (C) are provided SRH education material addressing cultural taboos in talking to adolescents about these topics and a radio program of the interactive drama (M-Res), then it may change their views and beliefs about contraceptive use, healthy relationships and how to communicate with their children - creating progressive gender and social norms (IO) because they will have the knowledge on these topcis, and may discuss it with other caregivers and peers after listening to the radio show. They also apply these comunication techniques with their children to improve their relationship (M-Rea).	IPT 6, community engagement

					to communicate personal expectations, preferences and limits<br>Normative beliefs: Believing that peers are not having sex until they feel ready;peers always use condoms when they have sex, peers should not pressure others to not use condoms, should not pressure each other not to use condoms; condom use is not a sign of a distrust in relationships.<br>Gender role norms: Believing that women have the right to make decisions about sex; women have the right to refuse sex; have the right to request the use of condoms and to refuse sex if condoms are not available; women in			

					transactional relationships have the right to refuse sex without condoms; using condoms does not emasculate men.			

Hemono2022	CyberRwanda	In facilitated model, the same implementation materials are provided as the self-service model with the addition of peer facilitators. The peer facilitators guide youth through structured sessions outlined in a CyberRwanda activity booklet.	High fertility rate, unmet need for family planning, high adolescent pregnancy	Comprehensive sexuality education through age-appropriate storytelling content about FP/RH STORIES: Fun and engaging narrative content in a web-comic format to engage all literacy levels in Kinyarwanda and English. Topics include career planning, goal-setting, puberty, contraception, gender-based violence, relationships, and consent.	The theory of planned behaviour (TPB) was used to inform the hypothesised impact pathway. It states that intention is central to behaviour change and that the primary influencers of intention are knowledge/beliefs and perceived behavioural control, both of which can shift subjective norms and thus influence intentions related to a particular behaviour. Actual control (juxtaposed with perceived behavioural control) can directly influence a behaviour by, for example, improving access by removing structural barriers.	contraceptive uptake, and increasing HIV testing.	33. If adolescents living in areas with high fertility rates and stigma around family planning (C) are provided a digital app with age and literacy-appropriate stories in a web-comic format on SRH either at select schools/youth centres or as self-service facilitated via peer educators (M-Res) then they may form more gender equitable beliefs, engage in safe sex behaviour, and access health services more often leading to reduction in adolescent pregnancy (O) because they engage with the information provided to them, which increases their knowledge on risk perception, and use of contraceptives, which may influence their intention towards safe sex practices. The peer educators may act as mentors who may motivate the adolescents to engage in safe sex. The private and secure aspect of using their own app will	Digital health

							also reduce the stigma around learning about SRH topics (M-Rea).	

		Direct-to-consumer platform - Technology - app use - networked tablets through two implementation models: self-service and facilitated.	Stigmatisation of FP/RH and discrimination from providers are frequently reported by young women and girls as reasons for not seeking services	Streamlined access to contraception and FP/ RH products at nearby pharmacies through a mobile ordering platform. SHOP: Youth can discreetly and privately order, purchase, and pick-up health products including condoms, emergency contraceptives, oral contraceptive pills, pads, ibuprofen, and paracetamol at the CyberRwanda pharmacy of their choice.<br>Pricing is transparent on the platform and users can choose from local pharmacies in the network.	We hypothesise that CyberRwanda's tailored narrative and content can influence behaviour change by increasing knowledge, perceived behavioural control, and access to FP/ RH products through online ordering and integration with youth-friendly pharmacies.	reducing early pregnancy	34. If adolescents living in areas with high fertility rates and stigma around family planning (C) are provided a digital app where they can discreetly and privately order, purchase, and pick-up health products including condoms, emergency contraceptives, oral contraceptive pills, and other medicines from a youth friendly pharmacy (M-Res) then they may increase their acccess to health services and products, they may increase the use of contraceptives which may lead to reduction in adolescent pregnancy (O) because adolescents will have access to safe and judgement free shops providing them services, they will have trust in the shops and health workers to give them correct information and the anonymous ordering creates autonomy and security for the participants	Digital health

							to access services without the threat of exposing their identity - this increases confidence in getting the care required (M-Rea).	

		Self-service model, schools and youth centres receive tablets, hotspots, marketing materials, and training on how to set up tablets in select in-school and youth centre locations for individual use.		App to LEARN: A robust library of questions with over 200 FAQs and a directory to help youth locate both public and private health facilities and pharmacies in their communities.		Students were interested in receiving information about FP/RH and reported high usage of the STORIES and LEARN features. Nearly 40% used Cyber-Rwanda to learn about contraceptives. IDIs revealed that ordering contraceptive products through the confidential SHOP feature was also of great interest to students; however, actual utilisation of this feature was low.	35. If adolescents living in areas with high fertility rates and stigma around family planning (C) are provided a digital app with age and literacy-appropriate FAQ section and a directory to locate youth friendly services either at select schools/youth centers or as self sevice facilitated via peer educators (M-Res) then they may engage in safe sex behavior, and acccess health services more often leading to reduction in adolescent pregnancy (O) because they engage with the information provided to them, which increases their knowledge on risk perception, planning to avoid risk, access and use of contraceptives, which may influence their intention towards safe sex practices. The private and secure aspect of using their own app will also reduce stigma around learning about SRH topics (M-Rea).	Digital health

		pharmacists and their staff are trained to support the provision of high-quality, youth-friendly care.						

Sandoy2016	RISE	teachers and CHAs/CHWs will be given a 5-day training	rural schools	Education: Contol arms- girls will be offered writing materials (exercise books, pencils and pens) as an incentive to participate.	Funding: Cash transfers target the poverty dimension, by making it somewhat less urgent for the guardians that the girl gets married and for the girl to receive gifts from a boyfriend. Increased schooling among adolescent girls is likely to empower them economically [61] and cognitively, and combined with postponed childbearing this can enable them to better protect the health of their children [17] and themselves and moreover increases the probability that their future children will complete secondary school	Socioeconomic inequalities, marriage, school attendance/ enrollment, use of contraceptives	36. If school-going girls (grade 7-8-9) and their families living in rural areas with common issues like school dropout, child marriage and adolescent pregnancy (C) are provided with economic support, consisting of a monthly cash transfer for the girl (ZMW 30), an annual cash grant to her parents/guardians (ZMW 350/year) and direct payment of school fees to school account (up to ZMW 500 per term) (M-Res) then the household poverty levels might decrease, girls may remain in school for longer, they may get married later and may postpone childbearing (O) because the families will have more financial resources to keep the girls in school for longer, they will have more resources to cater to other daily needs and therefore will not have to marry the girl to reduce their financial burden, the girls may have more	IPT 1, 3

							education which may increase her employability thereby reducing dependence on family and boyfriends. Girls may also be equipped with more knowledge and negotiation skills to communicate with parents and postpone their marriage and subsequent pregnancy/ with partners to postpone childbearing.	

		youth-friendly health services	medium school dropout rates, and adolescent marriage and childbearing are common.	Economic support: arms, girls and their parents/guardians will be offered economic support, consisting of a monthly cash transfer for the girl (ZMW 30), an annual cash grant to her parents/guardians (ZMW 350/year) and payment of school fees for girls who enrol in grade 8 and 9 (up to ZMW 500 per term).		Incidence of pregnancy and birth (beofre 16th/18th birthday) at different points in time		

				Community dialogue:(1) community and parent meetings employing a community dialogue approach in promoting supportive community norms around education for girls and postponement of early marriage and early childbearing	Interaction with parents: Meetings will be held to inform parents about the content of the youth club sessions.<br>Interaction with HCW: In addition, orientation meetings will be held to inform other healthcare workers in the catchment area of the schools about the project and the importance of providing youth-friendly health services.<br>Interaction with community: These meetings will be conducted using a dialogue approach and will discuss topics such as the value of education, and the risks and benefits of early childbearing.		37. If parents, health workers and the broader community members in rural areas with common issues like school dropout, child marriage and adolescent pregnancy (C) are involved in a community dialogue around education for girls, postponement of early marriage and early childbearing, the importance of providing youth-friendly health services and the risks and benefits of early childbearing (M-Res) then they may form more supportive/progressive community norms around educating girls, keeping them in school, marrying them later (O) because people in the community will have an increased understanding of girls lived realities and the impact on their health and wellbeing because of early marriage and child bearing - which will increase support for education, shift social and	IPT 6, community engagement

							gender norms to support girls and their future aspirations (M-Rea).	

				(2) establishment of youth clubs in order to provide comprehensive sexual and reproductive health education among in- and out-of-school adolescent girls and boys.	Interaction between youth: The meetings will include interactive discussions on education, early marriage, the risks of early pregnancy, gender roles, and sexual and reproductive health, including myths around modern contraceptives.		38. If both school-going and out-of-school adolescent girls living in rural areas with common issues like school dropout, child marriage and adolescent pregnancy (C) are involved in youth clubs that provide comprehensive sexual and reproductive health education including interactive discussions on education, early marriage, the risks of early pregnancy, gender roles, and sexual and reproductive health, including myths around modern contraceptives (M-Res) then they may recognise risky situations better, they may change their belief on contraceptive use and gender stereotypes, they may attend school for longer, **plan** and prevent engaging in unprotected sex and they may postpone marriage and childbearing (O) because interacting with their peers in a safe	IPT 5, 2, 4, 7 (life skill education + safe space + mentor)

							space may help them form social networks and trust, it may decrease their social isolation, it may lead to positive peer influence, they may find others to relate to and to discuss their issues with. More broadly, adolescents may have a supportive environment in their peer group, families and communities where they can discuss sensitive topics and have the power to make the right choice (M-Rea).	

George2020	Dreams	DREAMS was not uniformly rolled out across these districts.	high-HIV-burden districts	HIV Counselling and Testing (HTS) and linkages to care, pre-exposure prophylaxis (PrEP), barcoded card to access their HIV test results, voluntary medical male circumcision	reduce the HIV risk of men who are likely to be the sex partners of the AGYW.	Pregnancy was measured by asking AGYW if they have ever been pregnant	39. If vulnerable adolescent girls living in areas with high HIV burden (C) are provided linkage to youth friendly sexual reproductive health services, HIV counselling and testing with barcoded card to access results, and GBV prevention and post-violence care and support services (M-Res) then girls may increase HIV testing, but may not use contraceptives, reduce transactional sex and seek medical care in case of injuries (O) because the providers are not trained in how to discuss their needs in an appropriate and private manner, girls do not feel comfortable in accessing health services because of restrivtive nomrs around mobility and status of women and safety issues (M-Rea).	No effect CSMOC IPT 6
George2022		US government funded programme implementers, including community, faith-based, non-governmental organisations, and the South African government. Community engagement and advocacy with local stakeholders.	most at-risk populations and these areas were targeted for implementation of intervention	Linkage to sexual reproductive health (SRH) services, youth-friendly reproductive health care	empower AGYW to control their sexual health, understand girls' needs and not stigmatize them, trust in health system, staying STI free, efficacy (confidence with providers), access to services	HIV infection, STIs, condom use, age at first sex, pregnancy, number of partners, currently in school		

**George & Beckett2022**		Trained female staff only 20 to 25 years old	eThekwini District is home to the busiest port, main economic hub within the province of KZN - mostly rural	GBV prevention as well as post-violence care and support;	better understanding of gender-based violence, tools and resources to protect themselves and seek help when needed (agency), strategies to prevent GBV, improve participants' access to medical services - lead to early detection of violence	transactional sex, access to condoms		

Govender2022		Youth friendly health services	uMgungundlovu includes traditional settlements or farmlands, informal, rural and urban settlements.	Comprehensive sexuality education: Scripted Lesson Plans (SLPs) to strengthen the teaching of CSE in schools. SLPs are learner and teacher support materials (LTSMs) designed to aid educators and improve the effectiveness of CSE lessons. Condom promotion and provision, reduce risk of sex partners	To build and shape learners' understanding of concepts, content, values, and attitudes around sexuality and sexual behaviour, convincing male sexual partners to use condoms. Teachers equipped to answer questions on HIV infection. They feel comfortable talking about the topic. They had higher awareness about the topic. Interaction between students and teachers increased. Students feel comfortable with the existing teachers and trust them to provide correct information.	HIV testing and antiretroviral therapy uptake	40. If school-going vulnerable adolescent girls living in areas with high HIV burden (C) are provided comprehensive sexuality education in school by teachers (M-Res) even then they may not reduce transactional sex and sex with older men, increase use of contraceptives, HIV testing and may not delay sexual debut (O) because the teachers may not be trained, there may still be stigma and discomfort discussing these topics in class with an authority figure (M-Rea).<br><br>41. If teachers in schools in rural and highly-populated areas with high HIV burden (C) are provided scripted lesson plans including support material to strengthen their teaching methodology on CSE (M-Res) even then student's attendance, well-being and learning outcomes may not improve (O) because teachers are not trained to	No effect Material resource in school - social norms (new)

							use these materials, they may be over worked and do not have the time to engage with extra lessons on SRH topics which will not lead to positive social norm change in the school (M-Rea).	

Cawood2021				Social asset building: strengthen families with social protection (educational subsidies and combined socioeconomic approaches)	field staff relate to the participants interviewed, mitigating factors such as poverty, economically strengthen the families of AGYW and improve the ability of families to positively and effectively support AGYW		42. If families of vulnerable adolescent girls living in areas with high HIV burden and poverty (C) are provided educational subsidies (M-Res), even then girls may not stay in school for longer, they may not have better learning outcomes, and may not see reduction in adolescent pregnancy (O) because if subsidy goes directly to school the family would not see overall increase in funds and the families with limited financial resources may not want to invest in the education of their daughters (M-Rea).	NO effect IPT 1, 3 (economic incentive and financial training)
				Mobilize communities for HIV and violence prevention, parental or caregiver programs.	mobilize communities to change norms, create a supportive environment where adolescents can talk to their parents without judgment		43. If communities and caregivers in high HIV burden areas (C) are provided awareness and information on HIV and gender-based violence prevention (M-Res) even then the incidence of violence, HIV infection may not reduce (O) because community members and caregivers do not engage with these awarness	No effect Community engagement (new)

							activities. Theryby not leading to changes in restrictive social norms and not creacting supportive environment for adolescnt girls (M-Rea).	

Document 12: Programme theories and associated CMOCs

**Programme theories and underpinning context-mechanism-outcome configurations (CMOC)**	
**Programme theories for interventions delivered at the school setting targeting adolescents**	
**Programme Theory 1 – Supportive environment at school**<br>If schools (C1) are provided educational resources (e.g. laptops, internet, projector) for after-school sessions (M-Res1), teachers receive training on interactive teaching using digital remedial curricula (M-Res2) to provide underperforming female students (C2) with grade and subject-specific tutoring tailored to address their specific needs, and local female mentors from the community are assigned to schools to discuss gender and sexual reproductive health topics with female students (M-Res3)<br>Then, student attendance, psychosocial well-being and learning outcomes are likely to improve (IO)<br>Because using digital educational resources and needs-specific methods can enhance the learning environment by making learning more interactive and engaging, and cater to students' needs and learning styles (M-Rea1). Tailored tutoring by school teachers provides girls with individualised attention and assistance which increases their confidence and motivation to learn (M-Rea2). Discussing sensitive topics with mentors will help students voice their issues and engage with each other in a supportive environment (M-Rea3). Involving local female mentors also fosters a sense of community within the school environment, providing students with additional avenues for seeking help and aiding teachers with their workload (M-Rea4).<br>As a result, female students feel supported and motivated to learn (M-Rea5), the school observes positive social norm change (M-Rea6), and girls might continue with their education and find job opportunities, which might delay marriage and pregnancy (O).<br>**Supported by IPT: New PT**	
**CMOC**	**Evidence from interventions (For/Against PT)**
CMOC1 If schools in low-resource settings (C) are provided capacity building in the form of teacher training to better equip them to provide needs-based support in subjects like Maths and English, classroom equipment like laptops, internet and projector, and local mentor support to discuss gender-related inequality (M-Res) then the school will see overall gains in education and health outcomes (IO) because teachers will be more involved with students to discuss their problems in various subjects and mentors will be able to discuss topics of SRH which will reduce stigma on these topics. The students will be interested in learning and will regularly come to school to use the new equipment. This will create positive social norm changes in the school environment (M-Rea), which acts as a safe space for girls (M-Rea).	For (KGIS)
CMOC41 If teachers in schools in rural and highly-populated areas with high HIV burden (C) are provided scripted lesson plans including support material to strengthen their teaching methodology on CSE (M-Res), even then student's attendance, well-being and learning outcomes may not improve (O) because teachers are not trained to use these materials, they may be overworked and do not have the time to engage with extra lessons on SRH topics which will not lead to positive social norm change in the school (M-Rea).	Against (DREAMS)
CMOC2 If underperforming adolescent girls in school (C) are provided with need-specific remedial education using digital media and problem-based learning resources in both in-person and online formats (M-Res) - then school dropout will reduce (IO) - because new learning methods may lead to higher attention span, interest in class material, and retention of knowledge (M-Rea) which will improve the girl's performance at school. Families of girls performing well at school may, as a result, decide to keep them in school longer and may postpone child marriage, and this subsequently reduces adolescent pregnancy (O).	For (KGIS)
**Programme Theory 2 – Empowering girls through comprehensive sexual health education in safe spaces**<br>If school-going and out-of-school adolescents (C1) living in areas affected by gender inequities (e.g. high school dropout of girls, high prevalence of HIV/AIDS, early sexual debut for girls, child or early marriage and teenage pregnancy) (C2) are provided comprehensive sexual health education (including HIV/AIDS prevention, gender stereotypes, relative risk messaging, risky sexual	

behaviours and abstinence education, and in-class quiz or puzzle on these topics) using digital media (M-Res1) in a safe space (M-Res2) by trained facilitators (local female consultants or school teachers) (M-Res3)<br>Then, girls are likely to adopt less risky sexual behaviours such as using contraceptives, sex with fewer partners or similarly aged partners, postponing sex, and abstinence (IO)<br>Because discussion with trained facilitators and solving quizzes promote critical thinking and help process one's thoughts and beliefs (M-Rea1). Access to information delivered using visual aids increases knowledge retention and knowledge about modern contraceptives, and engagement with these materials helps with goal setting, decision making and planning for suitable outcomes (M-Rea2). Interaction with peers increases trust and develops negotiation skills and conformity to positive health behaviours (M-Rea3). Furthermore, trained facilitators can promote a gender-positive environment in the classroom because they have themselves critically reflected on SRH topics and are sensitised to discussing them (M-Rea4).<br>As a result, both school-going and out-of-school adolescents have more personal and relational control over their sexual health (M-Rea5). They feel supported and connected to their peers and teachers and make informed choices (M-Rea6), which might help prevent adolescent pregnancy (O).<br>**Supported by IPT: IPT 2, 3, 8**	
**CMOC**	**Evidence from interventions (For/Against PT)**
CMOC13 If school-going girls living in areas with high school dropout, HIV, early sexual debut, and teen pregnancy prevalence (C) are provided HIV prevention abstinence education in the classroom by trained teachers (male) (M-Res), then there may be a reduction in unprotected sex and subsequently in adolescent pregnancy (O) because teachers are well equipped to answer questions and feel comfortable doing so with girls in a classroom setting (M-Rea). This will increase interaction between teachers and students, leading to higher knowledge on the topic and improving trust and communication between them (M-Rea).	For (Sugar Daddy)
CMOC14 If school-going adolescents living in areas with high school dropout, HIV, early sexual debut, and teen pregnancy (C) prevalence are provided HIV prevention sexual health education using digital media and relative risk messaging in the classroom by trained female external consultants (M-Res) then they will have improved risky sexual behaviour, increase in contraceptive use and subsequent reduction in adolescent pregnancy (O) because the female consultant can connect with girls better (M-Rea) in comparison to male teachers both in terms of gender and power differential in classroom. Adolescents may feel more comfortable discussing sensitive topics (M-Rea) with someone new as it maintains privacy (M-Rea) in their immediate environment. The use of digital media may also help students visualise the information provided and increase information retention (M-Rea). Relative risk messaging promotes critical thinking about sexual decision-making and gives girls the power to choose (M-Rea) instead of abstinence-focused messaging, where they have only one choice (M-Rea).	For (Sugar Daddy)
CMOC15 If school-going adolescents living in areas with high school dropout, HIV, early sexual debut, and teen pregnancy (C) prevalence are provided sexual health education in a group setting in the classroom by trained teachers or consultants (M-Res), then they will have improved risky sexual behaviour, increase in contraceptive use and subsequent reduction in adolescent pregnancy (O) because interaction between peers will increase social comparison and conformity (M-Rea). If most peers think that getting pregnant before 18 is not suitable, that will influence others. On the other hand, if most peers think otherwise, that might lead to peer pressure (M-Rea) to indulge in risky practices. However, the presence of a trainer may control the spread of misinformation (M-Rea).	For (Sugar Daddy)
CMOC16 If school-going adolescents living in areas with high school dropout, HIV, early sexual debut, and teen pregnancy (C) prevalence are asked to take a test/quiz on personal and peer sexual health behaviours in a group setting in the classroom by trained teachers or consultants (M-Res) then they will have improved risky sexual behaviour, increase in contraceptive use and subsequent reduction in adolescent pregnancy (O) because the quiz will help them evaluate their thoughts and beliefs (M-Rea) on the topic. It will help them with goal setting and planning for suitable outcomes (M-Rea) and may lead to setting behaviours (M-Rea) on what not to do. Quiz being anonymous provides a low risk of stigma, and students can be true to what they think (M-Rea).	For (Sugar Daddy)
CMOC38 If both school-going and out-of-school adolescents living in rural areas with common issues like school dropout, child marriage and adolescent pregnancy (C) are involved in youth clubs that provide comprehensive sexual and reproductive health education including interactive discussions on education, early marriage, the risks of early pregnancy, gender roles, and sexual and reproductive health, including myths around modern contraceptives (M-Res) then they may	For (RISE)

recognise risky situations better, they may change their belief on contraceptive use and gender stereotypes, they may attend school for longer, plan and prevent engaging in unprotected sex and they may postpone marriage and childbearing (O) because interacting with their peers in a safe space may help them form social networks and trust, it may decrease their social isolation, it may lead to positive peer influence, they may find others to relate to and to discuss their issues with. More broadly, adolescents may have a supportive environment in their peer groups, families and communities where they can discuss sensitive topics and have the power to make the right choice (M-Rea).	
CMOC40 If school-going vulnerable adolescent girls living in areas with high HIV burden (C) are provided comprehensive sexuality education in school by teachers (M-Res), even then they may not reduce transactional sex and sex with older men, increase use of contraceptives, HIV testing and may not delay sexual debut (O) because the teachers may not be trained, there may still be stigma and discomfort discussing these topics in class with an authority figure (M-Rea).	Against (DREAMS)
**Programme Theory 3 – Empowering boys through gender norms-based sexuality and relationship education**<br>If school-going and out-of-school adolescent boys (C1) living in areas affected by gender inequities (e.g. high prevalence of HIV/AIDS, teenage pregnancy, and low access to sexual reproductive health services) (C2) are involved in group-based, context-specific activities like interactive comprehensive sexual health education (including television drama showcasing a young couple's journey with adolescent pregnancy, activities to identify gender roles and biases, and exercises to identify trusted adults to discuss SRH topics) (M-Res1)<br>Then, they are likely to challenge existing beliefs on gender roles and responsibilities, increase the use of contraceptives, delay the initiation of sexual intercourse, and subsequently lower the incidence of adolescent pregnancy (IO)<br>Because watching an interactive drama with peers would capture attention, evoke empathy, improve their understanding of social influences and norms around masculinity and make the information more relatable (M-Rea1). The gender-role activities would serve as behavioural modelling that would shape participants' perceptions and actions by applying knowledge on maintaining healthy and trusting relationships (M-Rea2). Discussions with peers foster dialogue, peer support, and reflection, potentially leading to shifts in attitudes, norms, and communication patterns within them (M-Rea3). Identifying and interacting with an adult of their choice would give them a sense of agency and spread positive gender norms (M-Rea4).<br>As a result, adolescent boys would be motivated to adopt positive subjective norms, understand its perceived benefits (M-Rea5) and have the confidence to change their sexual behaviour (M-Rea6), which might reduce adolescent pregnancy (O).<br>**Supported by IPT: New PT**	
**CMOC**	**Evidence from interventions (For/Against PT)**
CMOC30 If young boys both in school and out of school living in high HIV and adolescent pregnancy areas (C) are involved in a group-based, context-specific interactive drama showcasing a young couple's journey with adolescent pregnancy (M-Res) then it may change their ideas about gender roles and responsibilities, may lead to a change in beliefs about using contraceptives, may lead to delayed initiation of sexual intercourse and subsequently incidence of adolescent pregnancy (O) because they may gain knowledge and skills on how to use contraceptives, where to acquire it and how to avoid pregnancy; they will be able to perceive risky situations and plan accordingly to avoid pregnancy; and they will also know how to discuss these topics with their partners and peer to better negotiate for themselves (M-Rea).	For (If I were Thabo)
CMOC31 If young boys both in school and out of school living in high HIV and adolescent pregnancy areas (C) are involved in group-based, context-specific activities that involves identifying gender bias, roles and responsibilities and home-based activities to identify trusted adults to discuss SRH topics (M-Res) then it may change their ideas about gender roles and responsibilities, may lead to change in beliefs about using contraceptives, may lead to delayed initiation of sexual intercourse and subsequently incidence of adolescent pregnancy (O) because they may gain knowledge on how to maintain healthy and trusting relationships, they may gain skills to negotiate and communicate with partners about SRH topics, they may find advice from trusted adults, and they may have the confidence to apply this in their lives to prevent unwanted pregnancy (M-Rea).	For (If I were Thabo)

Programme theories for interventions delivered at the health facility setting for adolescents	
**Programme Theory 4 – Confidential youth-friendly health education and services**<br>If school-going adolescent girls and boys (C1) living in areas with high adolescent pregnancy (C2) are provided comprehensive sexual health education (M-Res1) in a safe group setting (M-Res2) by trained health providers (M-Res3) along with health services (e.g. HIV counselling and testing, information on pregnancy prevention and pregnancy testing, contraceptive counselling, menstrual health and personal hygiene promotion, and hypertension and obesity monitoring) at school via anonymous referral slips or access to services at youth-friendly health facilities outside of school (e.g. HIV/STI prevention, puberty, personal hygiene, menstrual health, and adolescent pregnancy prevention) (M-Res4)<br>Then, it is likely that their knowledge of sexual health topics and services increases, and they get regular tests and counselling sessions (IO)<br>Because discussing SRH topics with trained health providers and peers increases knowledge, creates a supportive environment, improves interpersonal relationships with peers, and reduces stigma and hesitation around these topics in a mixed-gender classroom (M-Rea1). Anonymous referral creates autonomy and security for adolescents (M-Rea2), which increases their confidence in the uptake of services, increases demand (M-Rea3) and aids risk reduction (M-Rea4). Access to youth-friendly health facilities removes barriers to accessing care and promotes proactive health-seeking behaviours (M-Rea5).<br>As a result, adolescents feel supported and safe in accessing health services (M-Rea6) and are motivated to make informed decisions about their sexual health (M-Rea7), which might reduce adolescent pregnancy (O).<br>**Supported by IPT: IPT 2, 3, 8**	
**CMOC**	**Evidence from interventions (For/Against PT)**
CMOC24 If adolescents (boys and girls) in school living in high pregnancy prevalence areas (C) are provided comprehensive sexual health education in class along with health fairs on school grounds and anonymous referral slips to a youth-friendly facility by trained health providers where they can get free testing, counselling and health monitoring (M-Res) then there is a reduction in unwanted adolescent pregnancy (O) because CSE and health fairs give access to information on school grounds in the presence of other peers who might also visit the sessions and stalls together - creating a supportive peer environment, and anonymous referral creates autonomy and security for the participants who want to access services without the threat of exposing their identity - this increases confidence in getting the care required translating into uptake of services (M-Rea).	For (CSE-Health Facility linkages)
CMOC25 If adolescents (boys and girls) in school living in high pregnancy prevalence areas (C) are provided comprehensive sexual health education in class along with access to health services outside of class at a youth-friendly facility where they can get free testing, counselling and health monitoring (M-Res), then there is a reduction in unwanted adolescent pregnancy (O) because CSE and access to free care may reduce stigma around the topic of risky behaviour - thus increasing trust in the system - that increasing uptake of available services. The provision of free health services may increase demand and aid risk reduction. Independent visits to the facility may create autonomy and security for the participants to access services without the threat of exposing their identity. (M-Rea).	For (CSE-Health Facility linkages)
CMOC12 If vulnerable adolescents at high risk of HIV, living in disadvantaged communities (C) are provided with HIV risk reduction specific health education, including information on HIV/AIDS, healthy communication, safe sex, decision making and substance abuse (M-Res), then their risk of adolescent pregnancy (O) may not reduce because the intervention only focuses on the individual and not their environment. The adolescents in disadvantaged communities may not have the power to use the skills learned in the course if negative community norms prevail (M-Rea). They might not have access to resources to buy health products because of a lack of funds and opportunities to gain employment (M-Rea) - thereby limiting the transformation of skills into positive outcomes.	Against (ES and HIV)
CMOC28 If adolescent and young boys and girls both in and out of school living in suburban areas with high cohabitation, early sexual debut and adolescent pregnancy (C) are provided with comprehensive sexual health education with digital media, educational resources, lectures and peer discussions (M-Res) then their coercing a partner into having sex decreases and use of contraceptives increases but no change in adolescent pregnancy (O) because comprehensive sexual health education increases knowledge about sexual health-related topics, and with new knowledge comes a sense of judgement to make healthy choices. Digital	Against (CSE)

media use, lectures and group discussions help with knowledge retention and inculcate a group learning environment and a sense of belonging with peers (M-Rea).	
**Programme Theory 5 – Fostering demand through digital health services and free health supplies**<br>If adolescent girls and boys (C1) living in areas with high prevalence of adolescent pregnancy, premarital sex, HIV/AIDS and sexual violence (C2) are provided with free access to a digital health platform with information on SRH (M-Res1), free access to youth-friendly health facilities with health services (e.g. counselling, HIV and pregnancy self-tests, family planning, medicines, contraceptives), and pharmacies selling health products (e.g. condoms, emergency contraceptives, oral contraceptive pills, and other medicines) (M-Res2)<br>Then, they are likely to increase the use of contraceptives, decrease unprotected sex, and increase testing for STIs, which subsequently lead to a reduction in adolescent pregnancy (IO, O)<br>Because adolescents can access health services and products without financial dependence on parents or partners (M-Rea1). Access to youth-friendly health providers gives non-judgmental care tailored to their unique needs, which will increase trust and support between the health providers and adolescents due to reduced shame and stigma (M-Rea2). They will have control over the type of information they access (M-Rea3), have privacy and anonymity in accessing services and information (M-Rea4), and have decision-making power to plan for suitable outcomes (M-Rea5). As a result, adolescents feel supported and safe in accessing health services (M-Rea6), are financially empowered to use them (M-Rea7), are motivated to act to improve their sexual and reproductive health and use contraceptives (M-Rea8) which might reduce adolescent pregnancy (O).<br>**Supported by IPT: New**	
**CMOC**	**Evidence from interventions (For/Against PT)**
CMOC7 If adolescent girls living in high teenage pregnancy, STI and HIV prevalent areas (C) are provided access to a digital health platform/app with information on SRH, free access to youth health facilities, and pharmacies selling health products (M-Res) then adolescents will increase their use of contraceptives, decrease unprotected sex, test for pregnancy and STI (O) because they have control over what type of information they access decreasing misinformation and misconception, how they access it and keep it anonymous through the app. They also have the choice to visit the health facility or pharmacy to get health products without being dependent on elders for funds. This will increase their overall sexual health agency and choice (M-Rea).	For (In their Hands)
CMOC23 If uneducated girls in communities with high sexual violence and high premarital sex prevalence (C) are provided health vouchers giving access to free health services at a youth-friendly facility (M-Res) then early pregnancy is delayed or prevented (O) because this may encourage them to access health services without financial dependence on parents, or partners, which in turn increases their decision-making power over their own sexual behaviour (M-Rea).	For (AGEP)
CMOC29 If adolescent and young boys and girls both in and out of school living in suburban areas with high cohabitation, early sexual debut and adolescent pregnancy (C) are provided reproductive health services and products (M-Res) then the use of contraceptives increases but no effect on adolescent pregnancy (O) because access to health services and counselling reinforces learning by application of skills learnt in the course, availability of free contraceptives and tests empowers participants to make decisions and use services accordingly (M-Rea).	For (CSE)
CMOC34 If adolescents living in areas with high fertility rates and stigma around family planning (C) are provided with a digital app where they can discreetly and privately order, purchase, and pick-up health products including condoms, emergency contraceptives, oral contraceptive pills, and other medicines from a youth friendly pharmacy (M-Res) then they may increase their access to health services and products, they may increase the use of contraceptives which may lead to reduction in adolescent pregnancy (O) because adolescents will have access to safe and judgement free shops providing them services, they will have trust in the shops and health workers to give them correct information and the anonymous ordering creates autonomy and security for the participants to access services without the threat of exposing their identity - this increases confidence in getting the care required (M-Rea).	For (CyberRwanda)
CMOC8 If adolescents (both boys and girls) living in high teenage pregnancy, STI and HIV prevalent areas (C) are provided free health products like counselling for contraceptives, HIV self-test kits, contraceptives and pregnancy testing at a youth-friendly health facility (M-Res) then they will increase the use of contraceptives and tests, decrease unprotected sex which will subsequently reduce adolescent pregnancy (O) because they will have access to information from	For (In their Hands)

a trusted health professional who understands their needs, does not stigmatize the subject, and keeps the information confidential. The free access also reduces financial dependence on parents and gives adolescents the power to make their own choices.	
CMOC35 If adolescents living in areas with high fertility rates and stigma around family planning (C) are provided a digital app with age and literacy-appropriate FAQ section and a directory to locate youth friendly services either at select schools/youth centres or as self-service facilitated via peer educators (M-Res) then they may engage in safe sex behaviour, and access health services more often leading to a reduction in adolescent pregnancy (O) because they engage with the information provided to them, which increases their knowledge on risk perception, planning to avoid risk, access and use of contraceptives, which may influence their intention towards safe sex practices. The private and secure aspect of using their own app will also reduce stigma around learning about SRH topics (M-Rea).	For (CyberRwanda)
CMOC33 If adolescents living in areas with high fertility rates and stigma around family planning (C) are provided a digital app with age and literacy-appropriate stories in a web-comic format on SRH either at select schools/youth centres or as self-service facilitated via peer educators (M-Res) then they may form more gender equitable beliefs, engage in safe sex behaviour, and access health services more often leading to reduction in adolescent pregnancy (O) because they engage with the information provided to them, which increases their knowledge on risk perception, and use of contraceptives, which may influence their intention towards safe sex practices. The peer educators may act as mentors who may motivate the adolescents to engage in safe sex. The private and secure aspect of using their own app will also reduce the stigma around learning about SRH topics (M-Rea).	For (CyberRwanda)
CMOC17 If adolescents (boys and girls) in peri urban communities with high prevalence of HIV (C) are provided access to trained health professional run, youth-friendly hubs with SRH services and products, digital CSE and referral to health facilities (M-Res) but amidst a public health emergency (COVID) (C) then their SRH knowledge, uptake of services, contraceptives and subsequent adolescent pregnancy may not reduce (O) because even though they have a safe space to discuss sensitive topics with professionals and peers, have positive reinforcement from the loyalty card to change behaviour and have independence from guardians in buying health products these spaces might be closed for extended periods and might be out of stock of various health products which may have led to loss of confidence and trust in the services provided (M-Rea).	Against (Yathu Yathu)
CMOC19 If the most vulnerable girls from lower-income backgrounds in both urban and rural areas (C) are provided comprehensive sexual and reproductive health education in a safe space in the community and free access to health services at youth-friendly facilities (M-Res), even then they might not have positive changes in sexual behaviour, GBV and adolescent pregnancy (O) because the most vulnerable girls might not be able to attend the sessions on SRH at the club due to travel restrictions from family, resource constraints to reach the place, responsibilities at home, job, sessions being not culturally appropriate, or they do not like the group setting due to privacy issues (M-Rea) thus leading them to not access the health service either.	Against (AGEP)
CMOC39 If vulnerable adolescent girls living in areas with high HIV burden (C) are provided linkage to youth-friendly sexual reproductive health services, HIV counselling and testing with barcoded card to access results, and GBV prevention and post-violence care and support services (M-Res) then girls may increase HIV testing, but may not use contraceptives, reduce transactional sex and seek medical care in case of injuries (O) because the providers are not trained to discuss their needs appropriately and privately, girls do not feel comfortable in accessing health services because of restrictive norms around mobility and status of women, and safety issues (M-Rea).	Against (DREAMS)
**Programme theories for interventions delivered at the community setting**	
**Targeting adolescent girls**	
**Programme Theory 6 – Empowering girls through employable life skills**<br>If vulnerable adolescent girls, both in-school and out-of-school (C1), living in areas with high gender disparities (e.g. low education and sexual knowledge and limited financial resources for girls) (C2) are provided face-to-face structured weekly life-skill sessions (e.g. on topics like health, finance, empowerment, computer training and nutrition) (M-Res1) in a safe space with peers (M-Res2), facilitated by trained local female role models /mentors (e.g. unmarried, young, educated, skilled women from the community with formal jobs) (M-Res3)	

Then, girls are likely to continue their education, engage in various employment activities, and postpone marriage and pregnancy (IO)<br>Because the life skill sessions equip adolescent girls with practical knowledge and skills like problem-solving, goal setting, decision-making and negotiation, which are essential for their personal and professional development (M-Rea1). The safe space with peers fosters a supportive environment for learning, sharing experiences, and building social connections and trust (M-Rea2). The mentors serve as relatable and aspirational figures for the girls, thus helping girls envision possibilities beyond traditional gender roles, motivating them to aspire to higher education and meaningful employment (M-Rea3).<br>As a result, girls are empowered (M-Rea4) and have higher human and social capital (M-Rea5), which equips them to overcome gender disparities, pursue employment opportunities, and delay marriage and pregnancy until they are ready (O).<br>**Supported by IPT: IPT 5, 7**	
**CMOC**	**Evidence from interventions (For/Against PT)**
CMOC3 If vulnerable adolescent girls both in school and out of school (C) are provided life skill sessions, including computer training and basic health services skills with female mentors from the community in a group setting (M-Res), then they may get involved in new job opportunities, may study more, postpone marriage and pregnancy (O) because the girls will experience low social isolation/ build social networks, gain problem-solving skills, gain technical know-how, get regular support from friends and mentors which will lead to an increase their human and social capital and will have improved decision making capacity (M-Rea).	For (KGIS)
CMOC22 If uneducated girls in communities with high sexual violence and high premarital sex prevalence (C) are provided trained mentor-led life skill training in a safe space with peers (M-Res), then early pregnancy is delayed or prevented (O) because discussing SRH topics with peers and mentor may increase knowledge and reduce the stigma associated with these topics and bring a sense of emotional support and shared experiences that further reduces isolation (M-Res). This may motivate them to set positive health and life goals for themselves and increase their decision-making power over their own sexual behaviour (M-Rea).	For (AGEP)
CMOC27 If vulnerable girls with low education, self-awareness, sexual knowledge and financial resources (C) are provided life skills training on a variety of health, finance and empowerment topics at a safe space in the community by a trained mentor (M-Res) then for girls who attend all the sessions adolescent pregnancy reduces (O) because clubs create a safe space for girls to talk about sensitive topics - which increases trust, belonging, reduction in isolation; interaction between girls and mentors also create healthy relationships for girls, mentor may act as a role model - increasing in aspirations, motivation, and emotional support; courses in club increase knowledge and skills - increase confidence and self-efficacy - increase in decision making power and judgment for healthy choices (M-Rea).	For (Sista2Sista)
CMOC11 If vulnerable adolescents at high risk of HIV, living in disadvantaged communities (C) are provided with economic strengthening courses on budgeting, saving and earning money (M-Res), then their knowledge of economic opportunities and financial knowledge may not increase (O) because they do not have an additional intervention component to apply this knowledge that will aid retention of knowledge (M-Rea). Also, as they live in disadvantaged communities without channels linking them to economic opportunities (M-Rea) - they will not find gainful employment. So, the knowledge gained will not convert into human capital (M-Rea) and, therefore, will not lead to changes in health outcomes.	Against (ES and HIV)
CMOC20 If the most vulnerable girls from lower-income backgrounds in both urban and rural areas (C) are provided life skill training by trained female mentors from the community in a safe space at the community even then they might not have positive changes in sexual behaviour, GBV and adolescent pregnancy (O) because the most vulnerable girls might not be able to attend the sessions at the club due to travel restrictions from family, resource constraints to reach the place, responsibilities at home, job, sessions are not appropriate for their needs, or they do not like the group setting due to privacy issues (M-Rea) thus limiting the translation of session into knowledge, aspiration and self-efficacy.	Against (AGEP)
**Programme Theory 7 - Empowering girls through economic support**<br>If in-school and out-of-school girls from poor families (C1) living in areas with high gender disparities (C2) are provided financial education or economic literacy (M-Res1) and financial assistance in the form of cash transfers, grants to parents or material incentives like payment of school fees (M-Res2)	

Then, they are likely to continue schooling, engage in livelihood opportunities, and postpone marriage (IO)<br>Because girls have decreased financial dependence on families and partners due to cash transfers (M-Rea1), the families have increased financial resources to keep girls in school (M-Rea2). Economic literacy provides girls with essential knowledge and skills to manage their finances effectively, and it empowers them to take control of their economic futures by imparting the skills to advocate for their rights, negotiate for fair wages, access financial services, and pursue entrepreneurial ventures or job opportunities (M-Rea3).<br>As a result, girls have increased economic and human capital (M-Rea4). They have higher control over available resources (M-Rea5) and decision-making capacity (M-Rea6), which might reduce adolescent pregnancy (O).<br>**Supported by IPT: IPT 1, 4, 7**	
**CMOC**	**Evidence from interventions (For/Against PT)**
CMOC4 If out-of-school girls (C) are provided financial education by a female mentor from the community combined with cash transfers for the most vulnerable girls (M-Res), then they may have more livelihood opportunities, which may lead to postponement of marriage and subsequent pregnancy (O) because they will have the job-relevant skills - learning by doing will help them retain and apply knowledge - which will increase their human capital. They will have decreased economic dependence on their families, which may increase their decision-making capacity. Regular meetings with mentors will also increase social connectedness and allow girls to form a social network, reducing social isolation and finding gainful employment (M-Rea).	For (KGIS)
CMOC36 If school-going girls (grade 7-8-9) and their families living in rural areas with common issues like school dropout, child marriage and adolescent pregnancy (C) are provided with economic support, consisting of a monthly cash transfer for the girl (ZMW 30), an annual cash grant to her parents/guardians (ZMW 350/year) and direct payment of school fees to school account (up to ZMW 500 per term) (M-Res) then the household poverty levels might decrease, girls may remain in school for longer, they may get married later and may postpone childbearing (O) because the families will have more financial resources to keep the girls in school for longer, they will have more resources to cater to other daily needs and therefore will not have to marry the girl to reduce their financial burden, the girls may have more education which may increase her employability thereby reducing dependence on family and boyfriends. Girls may also be equipped with more knowledge and negotiation skills to communicate with parents and postpone their marriage and subsequent pregnancy/ with partners to postpone childbearing.	For (RISE)
CMOC9 If adolescent girls belonging to poverty-stricken areas with low school retention, low formal employment opportunities and high incidence of child marriage and teen pregnancy (C) are provided unconditional cash transfers, then their families might have increased financial capacity to either send them to school or to invest in their employability or use it for other purposes - which may (in short term) or may not (long term) lead to decrease in child marriage and adolescent pregnancy because there is no clear pathway to ensure that the befit goes to the girls.	Against (SIHR)
CMOC21 If the most vulnerable girls from lower-income backgrounds in both urban and rural areas (C) are provided financial education by trained young mentors from the community in a group setting and access to a savings bank account, even then they might not have positive changes in knowledge on financial decision making, sexual behaviour, GBV and adolescent pregnancy (O) because they might not participate in the program or attend the session due to pre-existing barriers (see above) (M-Rea) even though these sessions and account are targeted to increase their economic asset.	Against (AGEP)
CMOC 42 If families of vulnerable adolescent girls living in areas with high HIV burden and poverty (C) are provided educational subsidies (M-Res), even then girls may not stay in school for longer, they may not have better learning outcomes, and may not see reduction in adolescent pregnancy (O) because if subsidy goes directly to school the family would not see overall increase in funds and the families with limited financial resources may not want to invest in the education of their daughters (M-Rea).	Against (DREAMS)

Targeting community members	
**Programme Theory 8 – Active involvement and Community Support for Adolescent Girls' Rights**<br>If stakeholders involved in childcare (including parents, health workers and broader community members) (C1) living in areas with high gender disparities (e.g. school dropout, high prevalence of child marriage and adolescent pregnancy) (C2) are engaged in community dialogue (M-Res1) and are provided health information using multimedia channels (radio, TV, in-person activities) on topics related to gender disparities (M-Res2) by trained community mobilisers (e.g. mentors, teachers) at a commonplace in the community (M-Res3)<br>Then, it is likely that school dropout would reduce and child marriage and adolescent pregnancy would decrease (IO, O)<br>Because there will be an increased understanding of the lived realities of girls in the community (M-Rea1). Community dialogue will promote ownership, buy-in, and commitment to implementing solutions within the community (M-Rea2). Restrictive social norms around these topics will be reduced by fostering a sense of collective responsibility and solidarity in the community (M-Rea3). There will be increased communication and trust between adolescent girls and their caregivers (M-Rea4), and girls will feel supported in voicing their opinions (M-Rea5). Disseminating health information to community members will enhance access to information and services and improve their participation in health-seeking for their children (M-rea6).<br>As a result, stakeholders strengthen interpersonal and family bonds (M-Rea7). They can better support their children, enabling children to strengthen their self-esteem, develop trust, and find ways to negotiate with caregivers (M-Rea8), which may lead to delay in child marriage, the continuation of school and reduced adolescent pregnancy (O).<br>**Supported by IPT: IPT 6**	
**CMOC**	**Evidence from interventions (For/Against PT)**
CMOC5 If families in communities with high child marriage prevalence (C) are engaged in group meetings with teachers and mentors on adverse effects of Child Marriage on adolescent girls, like impact on health, education, and employment opportunities (M-Res), then child marriages may reduce (O) because the people in the community will have an increased understanding of the lived realities of girls - which will increase support for education, skill building and shift social and gender norms to support girls and their future aspirations (M-Rea).	For (KGIS)
CMOC18 If peri-urban communities/families with a high prevalence of HIV (C) are provided community engagement activities by trained community mobilisers on SRH topics and services available for adolescents (M-Res) then social norms and stigma around these topics will reduce, leading to increased communication and support between parents and adolescents - further leading to positive sexual behaviour change (O). But in case of a public health emergency, these activities will be stopped, and a norm shift will not occur (M-Rea). Thus, leading to no impact on health.	For (Yathu Yathu)
CMOC26 If families in communities in high pregnancy districts (C) are provided with information and community engagement activities on health services available for adolescents at youth-friendly facilities (M-Res), then stigma and negative social norms around SRH will be reduced subsequently leading to uptake of services - risk reduction - and reduction adolescent pregnancy (O) because this will create a judgement-free atmosphere to access care for adolescents - increase in confidence, trust and sense of belonging for them in the community, and improve communication between parents and children (M-Rea).	For (CSE Health Facility Linkages)
CMOC37 If parents, health workers and the broader community members in rural areas with common issues like school dropout, child marriage and adolescent pregnancy (C) are involved in a community dialogue around education for girls, postponement of early marriage and early childbearing, the importance of providing youth-friendly health services and the risks and benefits of early childbearing (M-Res) then they may form more supportive/progressive community norms around educating girls, keeping them in school, marrying them later (O) because people in the community will have an increased understanding of girls lived realities and the impact on their health and wellbeing because of early marriage and child bearing - which will increase support for education, shift social and gender norms to support girls and their future aspirations (M-Rea).	For (RISE)
CMOC6 If families living in high teenage pregnancy, STI and HIV prevalent areas (C) are targeted for community sensitisation on adolescent SRH through a radio program on non-judgemental communication between parents and adolescents on sexual health (M-Res), then parents and adolescents will discuss SRH topics freely, adolescents' SRH knowledge will increase, and their contraceptive knowledge will increase subsequently leading to increase in contraceptive use and	For (In their Hands)

reduction in adolescent pregnancy (O) because adolescents will have a supportive environment in their families and communities where they can discuss sensitive topics, and have the power to make the right choice for themselves.	
CMOC32 If parents, caregivers and teachers (C) are provided SRH education material addressing cultural taboos in talking to adolescents about these topics and a radio program of the interactive drama (M-Res), then it may change their views and beliefs about contraceptive use, healthy relationships and how to communicate with their children - creating progressive gender and social norms (IO) because they will have the knowledge on these topics, and may discuss it with other caregivers and peers after listening to the radio show. They also apply these communication techniques with their children to improve their relationships (M-Rea).	For (If I were Thabo)
CMOC43 If communities and caregivers in high HIV burden areas (C) are provided awareness and information on HIV and gender-based violence prevention (M-Res), even then, the incidence of violence, and HIV infection may not reduce (O) because community members and caregivers do not engage with these awareness activities. Thereby not leading to changes in restrictive social norms and not creating supportive environment for adolescent girls (M-Rea).	Against (DREAMS)
